# A patchy theoretical model for the transmission dynamics of SARS-Cov-2 with optimal control

**DOI:** 10.1038/s41598-022-21553-1

**Published:** 2022-10-25

**Authors:** A. Mhlanga, T. V. Mupedza

**Affiliations:** 1 Department of Epidemiology and Biostatistics, Indiana University School of Public Health, Bloomington, IN USA; 2 Department of Mathematics, University of Zimbabwe, Box MP 167 Mount Pleasant, Harare, Zimbabwe

**Keywords:** Computational biology and bioinformatics, Mathematics and computing

## Abstract

Short-term human movements play a major part in the transmission and control of COVID-19, within and between countries. Such movements are necessary to be included in mathematical models that aim to assist in understanding the transmission dynamics of COVID-19. A two-patch basic mathematical model for COVID-19 was developed and analyzed, incorporating short-term human mobility. Here, we modeled the human mobility that depended on its epidemiological status, by the Lagrangian approach. A sharp threshold for disease dynamics known as the reproduction number was computed. Particularly, we portrayed that when the disease threshold is less than unity, the disease dies out and the disease persists when the reproduction number is greater than unity. Optimal control theory was also applied to the proposed model, with the aim of investigating the cost-effectiveness strategy. The findings were further investigated through the usage of the results from the cost objective functional, the average cost-effectiveness ratio (ACER), and then the infection averted ratio (IAR).

## Introduction

The recent outbreak of Coronavirus disease (COVID-19), a novel disease caused by severe acute respiratory coronavirus 2 (SARS-Cov-2), has resulted in a pandemic with an unmatched impact on societies all over the World. Several variants have been named by WHO and labelled as a variant of concern (VoC) or a variant of interest (VoI)^1^. The novel coronavirus can cause mild non-specific symptoms, including fever, dry cough, and fatigue (exhaustion). In more serious cases, it can develop into severe pneumonia, shivering (chills), pains, sore throat, difficulty breathing, headache, skin rashes, runny nose, taste loss, diarrhea, and fingers or toes dislocation^2^. According to the Centers for Disease Control and Prevention (CDC), COVID-19 has an incubation rate of around 14 days. The mean time for the symptoms to appear in a newly infected individual is about five days after contact. Rarely, symptoms appear as soon as two days after exposure^3^. COVID-19 is a highly infectious disease that can spread directly or indirectly from an infected individual to a healthy individual through the nose, eyes, and mouth by droplets created when an infectious individual produces respiratory droplets via coughing or sneezing^4,5^. Some studies have reported that an infected person with no symptoms can also transmit the virus^6–8^. Because of the many factors affecting the efficiency of environmental transmission, the relative risk of fomite transmission of SARS-CoV-2 is seen as compared with direct contact^9,10^. However, it is not clear what proportion of SARS-CoV-2 infections are acquired through indirect transmission, but they do exist. Various medical and non-medical methods have been used to curb the spread and prevalence of COVID-19. Different ways have been implemented to reduce transmission, such as wearing masks, keeping rooms well ventilated, avoiding crowds, physical distancing, coughing into a bent elbow or tissue, and cleaning your hands^11^. These various methods have been beneficial to some extent^12^, although COVID-19 vaccines have brought up much hope.

COVID-19 vaccines have reached billions of people worldwide, and the evidence is immense no matter which one you take. The vaccines offer life-saving protection against a disease that has taken millions of lives. However, the pandemic is far from over, and vaccines are our best bet for staying safe. It is worth stating that there is currently no trusted and highly effective treatment against COVID-19^13^. The few treatments available are only available to a few countries. Concerning COVID-19, recovered people can become susceptible again after a specific time, in particular, because of the appearance of new variants. Furthermore, waning of immunity towards COVID-19 has been reported, and it exists^14^.

While vaccine rates remain low, and COVID-19 continues to circulate in Africa. But the actual state of the pandemic is masked by a lack of testing. Although the continent accounts for nearly 17% of the world population, it accounts for around 1.8% of global tests. The pandemic is far from over in Africa, but there is also a funding gap in preparing for endemic COVID-19, which will require long-term investment in healthcare infrastructure^15^. A new challenge in the fight against COVID-19, which is a result of the implementation of intervention policies of governments, is hunger and poverty, especially in developing countries in sub-Saharan Africa like Zimbabwe, which lacks social security support.

Frequent visits between South Africa and Zimbabwe for trade and travel are a reality, including for individuals who want to support their families^16^. South Africa, Africa’s second most industrialized economy^17^, is Zimbabwe’s leading trade partner. Due to the socio-economic conditions in Zimbabwe, many people from Zimbabwe visit South Africa for shopping products which they resale back home for a living. Most recently, South Africans were flocking to Zimbabwe to access the vaccine, which they argued that Zimbabwe was rolling out reliably^18,19^. Even though trade in services involving proximity between suppliers and consumers, services involving travel, and temporary movement of people have been barred by almost all governments, it is worth noting that people still find their way via irregular means. The electric triple fence of razor-wire that marks the national limit between Zimbabwe and South Africa is cut regularly by rutted and worn tracks weaving through it. Even with lockdowns and border controls, little can be done to reduce the number of migrants between Zimbabwe and South Africa, even during the COVID-19 pandemic. Thus, the frequent movements between the two countries, which we may consider to be patched, are predominantly characterized by visitation (short-stays) as opposed to permanent migration. The movement of the asymptomatic infected and the exposed individuals during the incubation period contributes significantly to promoting the rapid spread of COVID-19. Contacts between infected and susceptible populations are the principal means for COVID-19 transmission, and the movement of people plays a crucial role in promoting their contacts^5^.

The low-risk and high-risk places are differentiated by their nutrition, health infrastructure, income, and health status variation, implying that they will also vary in terms of outcomes corresponding with the same number of cases^20^. In most high population density areas of Zimbabwe, poor working conditions and inadequate living space make social distancing very difficult as people in these high-risk areas migrate from one place to another in search of jobs and health care services in low-risk areas. People living in crowded places like Mbare often share water and sanitation services, making physical distancing and self-isolation difficult; this increases the risk of exposure to COVID-19^21^. Due to the strained economic conditions in Zimbabwe, most people in high-risk areas have few resources and are limited to bear the burden exerted by the pandemic^22^. Individuals from the high-risk areas visit those in the low-risk areas every day. Those from the low-risk regions also visit the high-risk areas daily since their businesses and workplaces are located within them. In Zimbabwe, it was noted that the low-risk areas were the COVID-19 hotspots^23^. The scenario is the same for most developing countries. Thus, the role of short-term disposal on the dynamics of COVID-19 between the two idealized interconnected, highly distinct communities might fuel or reduce COVID-19 prevalence. To make matters worse, a large share of the high-risk population depends on self-employment or the informal sector. Some are laborers in low-risk areas, which makes them vulnerable as they cannot afford health or social protection^24^. Many local and state governments turned to strict stay-at-home orders and shuttering businesses to defeat the virus transmission and save lives. However, there are long-term tremendous economic and social effects. In a quest to successfully control COVID-19, this has caused nations to vaccinate their populations while using non-pharmaceutical strategies^25^. It is worth noting that enacting strict policy measures does not necessarily mean a reduction in mobility but rather depends on issues concerning compliance and enactment of policies.

At a worldwide level, the breakout of unexpected infectious diseases causes several impacts on the economic sector. For example, the first visible impact could be preventive and containment measures taken by governments using their restricted resources. New mathematical models for COVID-19 that can be used and have been used for simulations to flatten the curve, predict the future behavior of its spread, and make decisions to control the transmission of COVID-19 have been developed and analyzed, such as^26–34^. There are models which have applied optimal control to understand the dynamics of COVID-19, see^35–45^. Aldida et al.^35^ proposed an optimal control mathematical model in which they successfully showed how community awareness played a crucial role in determining the success of COVID-19 eradication. A data-driven optimal control approach that integrated the reported partial data with the epidemic dynamics for COVID-19 was presented by Liu, and Tian^36^. In their work, they used the basic SEIR model to forecast the outbreak’s evolution over a relatively short period and provide scheduled controls of the epidemic. Silva et al.^37^ developed a mathematical model in which they applied optimal control theory to maximize the number of people returning to “normal life” while minimizing the number of active COVID-19 infected individuals with minimal economic costs while warranting a low level of hospitalizations. Gatyeni et al.^38^ formulated an optimal control problem in which they found out that joint implementation of effective mask usage, physical distancing, and active screening and testing are effective measures in the control of COVID-19. The effects of quarantine, isolation and public health education strategies using mathematical modeling and optimal control approach to ascertain their contributions to the dynamic transmission of COVID-19 were studied by Madubueze et al.^40^. Most recently, Arruda et al.^39^ derived optimal mitigation strategies over a prescribed time horizon under a more realistic framework that did not imply perennial immunity and a single strain. This study is of great relevance as it seeks to find cost-effective ways of halting the spread of COVID-19 by providing minimal economic and social disruptions that may stop an impending catastrophic rapture. As we seek to understand the effectiveness of mobility and activity restrictions in containing the outbreak of COVID-19, we will also benefit as it will equip us better to face future disease outbreaks. The effectiveness of optimal control in epidemiology is familiar: while mathematical modeling of epidemic diseases has shown that combinations of isolation, quarantine, vaccination, and/or treatment are often necessary to suppress or mitigate an epidemic disease, optimal control theory can play a paramount role by informing us on how they should be managed, by giving the right times for intervention and the right amounts^46,47^. Many researchers have successfully applied optimal control theory to proffer intervention measures that helped understand the dynamics of diseases such as Ebola, Zika, HIV, and TB using various mathematical models (see, e.g.^48–50^). In some of these studies, optimal control techniques are developed and applied to understand how the spread of these diseases may be controlled, e.g., through education campaigns, vaccination, treatment, quarantine, or isolation, with optimal implementation costs.

A number of mathematical models have looked into mobility restrictions on the control of epidemics, see^20,51–57^. The methods entail applying a Lagrangian approach, which uses a residence time matrix to track mobility between two communities. The importance of epidemiological frameworks that follow a Lagrangian approach is further explained in^51,58^. Of all the manuscripts, only one paper looked into the impact of mobility restrictions on the transmission dynamics of COVID-19^51^. The paper focused on the impact of mobility restrictions between areas with different infection risk levels. The main result of the manuscript was that mobility restrictions between two patches (low and high-risk areas) do not always reduce the final epidemic size. They underlined the point that restrictions could sometimes provide negative results. The result obtained by Espinoza et al.^51^ is very much epidemiological in that it only focuses on the impact of mobility restrictions on the final population size of the epidemic, and it does not speak to the effects of the efficiency of mobility restrictions as a disease control measure. It is important to highlight that the disease dynamics are sensitive to the cost of the disease. Hence it is vital also to investigate the cost of the disease. Suppose we are not to scrutinize the economic costs of disease. In that case, that implies that mobility restrictions should increase until the global final epidemic size is reduced to a minimum. The low-income countries and communities within developed countries were the most affected in trying to control the disease. This was partly due to the poor state of their public health infrastructures.

Driven by the frequent movements of individuals between Zimbabwe and South Africa or within Zimbabwean communities, we develop a mathematical model to describe the impact of short-term human activities on the persistence of COVID-19. These movements can happen in any country or region; hence they are not unique to South Africa and Zimbabwe. Thus, the study applies to other places/countries. To the best of our knowledge as authors, no mathematical model has looked into the transmission dynamics of COVID-19, utilizing the Lagrangian approach to discussing the short-term time residences. Furthermore, being conscious of the costs incurred in the mitigation strategies used. It is against this background that our study finds relevance and motivation. Mobility restrictions, such as border closures, trade, travel bans, quarantines, and extreme cases *cordon sanitaires*, are some of the most applied control measures to curb the spread of infectious diseases. In this manuscript, we seek to develop a two-patch mathematical model in which the two distinct communities are connected through the movement of individuals. Time spent by the people within each respective community/patch is modeled and applied to understand COVID-19 transmission dynamics under multiple mobility regimes. For more on the applications and further reading on the dispersal of individuals via a Lagrangian approach, see^51,58^. With the aid of optimal control theory, we investigate the impact of some control variables on the transmission dynamics of COVID-19. We also determine the cost-effectiveness analysis of these control variables using cost-effectiveness analysis. Our mathematical model is also based on a susceptible-exposed-infectious-recovered type of model, which also includes the aspect of the waning of the vaccine, which most COVID-19 models have not considered.

The paper is structured as follows. The COVID-19 mathematical model is formulated in “Formulation of the model” and analytical results of the model are presented in “Model analysis”. In “Formulation and analysis of the optimal control”, optimal control theory has been applied to the model formulated in “Formulation of the model”. Simulation results and projection profiles of COVID-19 are presented in “Numerical simulations”. Section “Cost-effectiveness analysis”, we present the Cost-effectiveness analysis and the Summary and concluding remarks round up the paper.

## Formulation of the model

A mathematical model is presented to understand the dynamics of COVID-19 within a domain characterized by two patches of heterogeneous risk. Precisely, it models the time spent by human beings in each community through a residency time matrix. Within a scenario of only two communities, it is assumed that time spent in one community is spent in the other. Individuals either spend their time in a single community or divide their time between two communities. Let $N_{i}(t)$ be the total population of individuals in patch *i* at time $t,~i=1,2$. Assuming that individuals of patch *i* spend $p_{ij} \in [0,1]$ in patch *j*, with $\sum _{j=1}^{2} p_{ij} = 1$, for each *i*. Thus, on average individuals of patch 1 spends, the proportion $p_{11}$ of their time of residency in patch 1 and the proportion $p_{12}$ of their time in patch 2 such that $p_{11} + p_{12} = 1$. The same applies to the individuals in patch 2. Individuals of patch 2 spend the proportion $p_{22}$ of their time in patch 2 and $p_{21}=1-p_{22}$ in patch 1. Thus, the effective population in patch 1 and patch 2 are given by

$$\begin{aligned} p_{11}N_{1} + p_{21}N_{2}~~~\text{ and } ~~~ p_{12}N_{1} + p_{22}N_{2} \end{aligned}$$

respectively.

The susceptible individuals of patch 1 $(S_{1})$ could be infected through direct contact in patch 1 (if currently in patch 1, that is, $p_{11}S_{1}$) or in patch 2 (if currently in patch 2, that is, $p_{12}S_{1}$). It follows from the above discussion that the effective proportion of infectious individuals in patch 1 is

$$\begin{aligned} \dfrac{p_{11}I_{1} + p_{21}I_{2}}{p_{11}N_{1} + p_{21}N_{2}}. \end{aligned}$$

By the same, it follows that the effective proportion of infectious individuals in patch 2 is given by

$$\begin{aligned} \dfrac{p_{12}I_{1} + p_{22}I_{2}}{p_{12}N_{1} + p_{22}N_{2}}. \end{aligned}$$

The following system of ordinary differential equations (ODE’s) represents the two patch COVID-19 model:

1 $$\begin{aligned} \left\{ \begin{array}{llll} S_{i}' = b_{i} -\Lambda _{i}S_{i} - \mu _{i} S_{i} + \kappa _{i} R_{i}, \\ E_{i}' = \Lambda _{i} S_{i} - (\mu _{i} + \omega _{i})E_{i}, \\ I_{i}' = \omega _{i} E_{i} - (\mu _{i} + \gamma _{i} + v_{i}) I_{i}, \\ R_{i}' = \gamma _{i} I_{i} - (\mu _{i} + \kappa _{i}) R_{i}, \\ W_{i}' = \eta _{i} I_{i} - r_{i}W_{i},~~~i=1,2, \end{array} \right. \end{aligned}$$

where

$$\begin{aligned} \Lambda _{i} = B_{i}p_{ii}\dfrac{W_{i}}{K_{i}} + \displaystyle \sum _{j=1}^{2} \beta _{j} p_{ij} \dfrac{\sum _{k=1}^{2} p_{kj} I_{k}}{\sum _{k=1}^{2} p_{kj} N_{k}}. \end{aligned}$$

**Figure 1:**
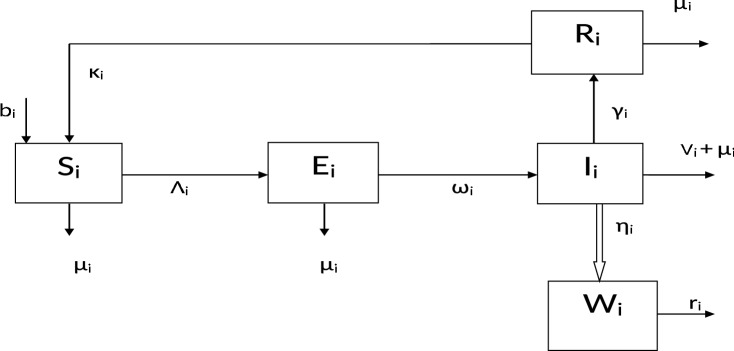
Model flow diagram.

The model partitions the human population at time *t* into the susceptible $S_{i}(t),$ exposed $E_{i}(t),$ infectious $I_{i}(t)$ and the recovered $R_{i}(t)$. The class $I_{i}$ represents the infectious pre-symptomatic $(I_{i}^p)$, infectious asymptomatic $(I_{i}^a)$ and the infectious symptomatic $(I_{i}^s)$. Thus, $I_{i} = I_{i}^p + I_{i}^a + I_{i}^s$ and we will make use of an *S*, *E*, *I*, *R*, *S* type of model. $b_{i}$ represents the recruitment rate of the individuals. $\mu _{i}$ represents the natural death rate, whereas the death due to COVID-19 is denoted by $v_{i}$. $\beta _{i}$ denotes the disease transmission rate by the infected individuals, and $B_{i}$ denotes the indirect infection from the COVID-19 virus in the environment to the susceptible individuals; $\omega _{i}$ is the progression rate from the exposed class to the infectious class. The recovery rate of the infected individuals is at rate $\gamma _{i}$. Parameter $\eta _{i}$ represents the rate of virus spread to the environment by the infected individuals. The death of pathogens in the environment is at a rate $r_{i}$. The pathogen vector in the environment is denoted as $W_{i}(t)$ whereas $K_{i}$ is the number or quantity of pathogens present during the interaction of human beings at time *t*. We assume that there is no permanent immunity. Hence, the recovered individuals become susceptible again at rate $\kappa _{i}$. Hence for the human population, we have $N_{i}(t) = S_{i}(t) + E_{i}(t) + I_{i}(t) + R_{i}(t)$ for $i=1,2.$ The model flow diagram is depicted in Fig. 1.

### Boundedness and positivity of solutions

It can be ascertained that the zone of biological interest

2 $$\begin{aligned} {\mathcal {G}} = \left\{ (S_{i},E_{i},I_{i},R_{i},W_{i}) \in {\mathbb {R}}^{10}_{+} \mid S_{i} + E_{i} + I_{i} + R_{i} \le \dfrac{b_{i}}{\mu _{i} + v_{i}}, W_{i} \le \dfrac{\eta _{i} b_{i}}{r_{i}(\mu _{i} + v_{i})}, ~~i=1,2 \right\} \end{aligned}$$

is attracting and positively invariant with respect to model (1).

## Model analysis

### The disease-free equilibrium and basic reproduction number

System (1) has an apparent equilibrium ${\mathcal {E}}^{0} = (S_{1}^{0},0,0,0,0,S_{2}^{0},0,0,0,0)$ when there is no disease, where

3 $$\begin{aligned} \left\{ \begin{array}{llll} S_{1}^{0} = \dfrac{b_{1}}{\mu _{1}}, \\ S_{2}^{0} = \dfrac{b_{2}}{\mu _{2}}. \end{array} \right. \end{aligned}$$

By utilizing the next generation approach, as outlined in Van den Driessche and Watmough^59^, we derive the basic reproduction number, ${\mathcal {R}}_{0}.$ Following^59^, the non-negative matrix *F* and the non-singular matrix *V* for the new infection terms and the remaining transfer terms are respectively given at the disease-free equilibrium by

4 $$\begin{aligned} F = \begin{bmatrix} 0 &{} M_{1} &{} M_{2} &{} 0 &{} M_{3} &{} 0 \\ 0 &{} 0 &{} 0 &{} 0 &{} 0 &{} 0 \\ 0 &{} 0 &{} 0 &{} 0 &{} 0 &{} 0 \\ 0 &{} M_{4} &{} 0 &{} 0 &{} M_{5} &{} M_{6} \\ 0 &{} 0 &{} 0 &{} 0 &{} 0 &{} 0 \\ 0 &{} 0 &{} 0 &{} 0 &{} 0 &{} 0 \\ \end{bmatrix}~\text{ and }~~ V = \begin{bmatrix} a_{1} &{} 0 &{} 0 &{} 0 &{} 0 &{} 0 \\ -\omega _{1} &{} \phi _{1} &{} 0 &{} 0 &{} 0 &{} 0 \\ 0 &{} -\eta _{1} &{} r_{1} &{} 0 &{} 0 &{} 0 \\ 0 &{} 0 &{} 0 &{} a_{2} &{} 0 &{} 0 \\ 0 &{} 0 &{} 0 &{} - \omega _{2} &{} \phi _{2} &{} 0 \\ 0 &{} 0 &{} 0 &{} 0 &{} -\eta _{2} &{} r_{2} \\ \end{bmatrix}. \end{aligned}$$

The spectral radius of the next generation matrix $FV^{-1},$ gives us the reproduction number ${\mathcal {R}}_{0}$ of system (1), given by

5 $$\begin{aligned} {\mathcal {R}}_{0} = \dfrac{1}{2}\left( \dfrac{(M_{1}r_{1} + M_{2}\eta _{1})\omega _{1}}{a_{1}r_{1}\phi _{1}} + \dfrac{(M_{5}r_{2} + M_{6}\eta _{2})\omega _{2}}{a_{2}r_{2}\phi _{2}} + \sqrt{\begin{matrix} &{} \left( \dfrac{(M_{1}r_{1} + M_{2}\eta _{1})\omega _{1}}{a_{1}r_{1}\phi _{1}}\right) ^{2} + \left( \dfrac{(M_{5}r_{2} + M_{6}\eta _{2})\omega _{2}}{a_{2}r_{2}\phi _{2}} \right) ^{2} \\ \\ &{}- 2 \left( \dfrac{(M_{1}r_{1} + M_{2}\eta _{1})\omega _{1}}{a_{1}r_{1}\phi _{1}}\right) \left( \dfrac{(M_{5}r_{2} + M_{6}\eta _{2})\omega _{2}}{a_{2}r_{2}\phi _{2}} \right) \\ &{}+ 4 \left( \dfrac{\omega _{1}M_{3}}{a_{1}\phi _{1}} \right) \left( \dfrac{\omega _{2}M_{4}}{a_{2}\phi _{2}} \right) \end{matrix}}\right) , \end{aligned}$$

where

6 $$\begin{aligned} M_{1}= & {} \dfrac{p_{11}^{2}\beta _{1}N_{1}}{p_{11}N_{1} + p_{21}N_{2}} + \dfrac{p_{12}^{2}\beta _{2}N_{1}}{p_{12}N_{1} + p_{22}N_{2}},~~M_{2} = B_{1}p_{11}\dfrac{N_{1}}{K_{1}},~~ M_{3} = \dfrac{p_{11}p_{21}\beta _{1}N_{1}}{p_{11}N_{1} + p_{12}N_{2}} + \dfrac{p_{12}p_{22}\beta _{2}N_{1}}{p_{12}N_{1} + p_{22}N_{2}}, \nonumber \\ M_{4}= & {} \dfrac{p_{11}p_{21}\beta _{1}N_{2}}{p_{11}N_{1} + p_{12}N_{2}} + \dfrac{p_{12}p_{22}\beta _{2}N_{2}}{p_{12}N_{1} + p_{22}N_{2}},~~ M_{5} = \dfrac{p_{21}^{2}\beta _{1}N_{2}}{p_{11}N_{1} + p_{21}N_{2}} + \dfrac{p_{22}^{2}\beta _{2}N_{2}}{p_{12}N_{1} + p_{22}N_{2}}, ~~ M_{6} = B_{2}p_{22}\dfrac{N_{2}}{K_{2}}, \nonumber \\ a_{1}= & {} \mu _{1} + \omega _{1},~~a_{2} = \mu _{2} + \omega _{2},~~\phi _{1}=\mu _{1} + v_{1} + \gamma _{1}, ~~\phi _{2}=\mu _{2} + v_{2} + \gamma _{2}. \end{aligned}$$

Following Theorem 2 in van den Driessche and Watmough^59^, the following result is established.

#### Theorem 1

If ${\mathcal {R}}_{0} < 1,$ the disease free equilibrium (DFE) ${\mathcal {E}}^{0}$ is locally asymptotically stable and unstable otherwise.

Additionally, a robust outcome with regard to the global dynamics of the DFE can be confirmed. We make use of the Lyapunov functions approach^60–62^ in analyzing the global asymptotic stability.

#### Theorem 2

If ${\mathcal {R}}_{0} \le 1$, the DFE is globally asymptotically stable in ${\mathcal {G}}$. If ${\mathcal {R}}_{0} > 1$, the system is uniformly persistent.

Proof of Theorem 2 is outlined in Online Appendix A. The result established in Theorem 2 portrays that ${\mathcal {R}}_{0} = 1$ is an acute threshold for disease dynamics: the disease dies out when ${\mathcal {R}}_{0} \le 1,$ persists when ${\mathcal {R}}_{0} > 1.$ Biologically if a system is uniformly persistent, it means that the disease persists for a long period. We now explore uniform persistence, and we assert the following theorem.

#### Theorem 3

If ${\mathcal {R}}_{0} > 1,$ system (1) is uniformly persistent, namely, there exists a constant $\xi > 0$ such that

$$\begin{aligned} \begin{array}{llll} \displaystyle { \lim _{t\rightarrow \infty } \inf S_{i}(t)> \xi ,~~\lim _{t\rightarrow \infty } \inf E_{i}(t)> \xi ,~~\lim _{t\rightarrow \infty } \inf I_{i}(t)> \xi ,~~\lim _{t\rightarrow \infty } \inf R_{i}(t)> \xi ,~~ \lim _{t\rightarrow \infty } \inf W_{i}(t) > \xi } \\ \end{array} \end{aligned}$$

for any initial conditions satisfying

$$\begin{aligned} S_{i}(0)\ge 0,~E_{i}(0)\ge 0,~I_{i}(0)\ge 0,~R_{i}(0)\ge 0,~W_{i}(0)\ge 0. \end{aligned}$$

The proof for Theorem 3 is presented in Online Appendix B.

### Analysis of the reproduction number

Table 1 outlines a summary of the model parameters and their baseline values. Due to the complexity of the model, fitting the model to data was a challenge because of the short-time residences. Furthermore, models such as this require tuning of the parameters since connecting empirical observations to parameters is complex. Therefore, some parameters are assumed inside the realistic ranges for illustrative purposes. Finally, the parameter values align with the historical lineage at the beginning of the COVID-19 pandemic.

**Table 1:** Model parameters and their baseline values.

Parameter	Definition	Baseline values	References
$\beta _{1}$	Rate of transmission due to the infected individuals	0.0115	^74^
$B_{1}$	Rate of transmission due to the environment	0.00414	^74^
$\beta _{2}$	Rate of transmission due to the infected individuals	$\alpha \beta _{1}$	Assumed
$B_{2}$	Rate of transmission due to the environment	$\alpha B_{1}$	Assumed
$\alpha$	Modification factor, for high risk community	$\alpha > 1$	Assumed
$b_{i}$	Birth rate of the human population	0.00018	^74^
$\mu _{i}$	Natural death rate	4.563 $\times ~10^{-5}$	^74^
$v_{i}$	Death due to COVID-19	0.0018	^74^
$\omega _{i}$	Progression rate from *E* to *I* class	0.09	^74^
$\eta _{i}$	Shedding rate by the infected individuals	0.075	^74^
$r_{i}$	Life expectancy of the pathogens in the environment	0.7124	^74^
$\gamma _{i}$	Recovery rate	(0.0169607 - 0.061)	^74^

The time unit is a day.

To understand the impact of short-term human mobility on the production of new infections, we calculate the values of ${\mathcal {R}}_{0}$ using the residence-time matrix in Table 2.

**Table 2:** Parameter values in a residence-time matrix.

**Coupling intensity**	Weak coupling	Strong coupling
Symmetric coupling	$p_{11}=0.99, p_{12}=0.01, p_{21}=0.01,$	$p_{11}=0.7, p_{12}=0.3, p_{21}=0.3,$
	$p_{22}=0.99 \,\,({{{\mathcal {R}}}_{0}=3.96})$	$p_{22}=0.7\,\, ({{{\mathcal {R}}}_{0}=2.82})$
Asymmetric coupling	$p_{11}=0.9, p_{12}=0.1, p_{21}=0.001,$	$p_{11}=0.7, p_{12}=0.3, p_{21}=0.001,$
	$p_{22}=0.999 \,\,({{{\mathcal {R}}}}_{0}=3.84)$	$p_{22}=0.999 \,\,({{{\mathcal {R}}}_{0}=3.79})$

The residence-time matrix organization includes the mobility patterns and the coupling intensity. The values in Table 2 have been taken from^57^, and in Table 1, only the upper bound for the recovery rate was taken from^74^. In this case, the weak coupling means that most individuals reside in their patch, while strong coupling means that some individuals temporarily go to the other patch. Mobility patterns constitute the symmetry of human movement between the two patches. For instance, we say it is symmetric if the proportion of humans moving from patch *i* to patch *j* is the same as for patch *j* to patch *i*. If the time spent for each proportion becomes more asymmetric, it becomes asymmetric mobility.

Using the values defined in Tables 1 and 2, we computed the reproduction numbers for Patch 1 and 2 without human mobility. We obtained ${\mathcal {R}}_{01}= 1.2069$ and ${\mathcal {R}}_{02} = 0.42138$. We can see that Table 2 suggests that the basic reproduction number is always high when the coupling intensity is weak. That is when most individuals stay in their patch. Additionally, we observe that the highest value of the reproduction number happens when there is symmetric mobility. Furthermore, we can observe that whenever there is human mobility, the human transmission risk increases globally instead of locally; for instance, in the absence of human mobility, we expect COVID-19 to die off in Patch 1. We note that the reproduction number is also a bit lower under the strong asymmetric coupling, that is when residents of patch 1 (high risk) spend large amounts of time in patch 2 (low risk); this makes sense as it exposes them to a more favorable, ${\mathcal {R}}_{0}$. We can see that the results in Table 2 show that when human mobility increases, the basic reproduction number decreases. Nevertheless, it will never drop below 1 for all cases illustrated in Table 2. Hence, under our assumption, we can conclude that effective human COVID-19 control will always be difficult to attain whenever there is human movement.

### Endemic equilibrium point

To examine the endemic equilibrium point’s local stability, we will use the Centre Manifold Theory, and the following result is established.

#### Theorem 4

The endemic equilibrium point ${\mathcal {E}}^{*}$ is locally asymptotically stable for ${\mathcal {R}}_{0} > 1$ and sufficiently close to 1.

Proof of Theorem 4 is presented in Online Appendix C.

## Formulation and analysis of the optimal control

In this section, we construct an optimal control problem to implement effective patch-precise control measures considering different coupling intensities. The two-patch COVID-19 model is adjusted by integrating the patch-specific control functions $(1-u_{i}(t))$ into the forces of infection for both patches 1 and 2 in model system (1). The control effort $u_{i}(t)$ models optimal educational campaigns. The educational campaigns are disseminated through all the available platforms, including social media. In this case, educational campaigns encourage those not infected to have some protective behaviors. Under COVID-19 preventive measures, the control efforts may include increasing public confidence in COVID-19 vaccines while reinforcing basic prevention measures, including mask-wearing and social distancing. It is vital to note that the infection risk will be reduced if the educational campaigns are strengthened with higher efficacy. Simultaneously, we wish to minimize the costs in attaining this. During the COVID-19 pandemic, much concern has been conveyed about the economic costs of disease control within the respective nations and the world. Hence, disease control and the associated costs are vital to recognizing an optimal public health response. Thus, in this section and “Numerical simulations”, we will focus on the state in which mobility restrictions may be efficient in minimizing the complete number of secondary infections within the two patches at minimum costs. Making use of the same variables and parameter names as presented before, our model system with the time-dependent controls is now given by

7 $$\begin{aligned} \left\{ \begin{array}{llll} S_{i}' = b_{i} - (1-u_{i}) \Lambda _{i}S_{i} - \mu _{i} S_{i} + \kappa _i R_{i}, \\ E_{i}' = (1-u_{i}) \Lambda _{i} S_{i} - (\mu _{i} + \omega _{i})E_{i}, \\ I_{i}' = \omega _{i} E_{i} - (\mu _{i} + \gamma _{i} + v_{i}) I_{i}, \\ R_{i}' = \gamma _{i} I_{i} - (\mu _{i} + \kappa _{i}) R_{i}, \\ W_{i}' = \eta _{i} I_{i} - r_{i}W_{i},~~~i=1,2. \end{array} \right. \end{aligned}$$

Our aim is to minimize the numbers of clinically infected individuals over a finite time horizon [0, *T*] at minimal costs in each respective patch. Mathematically, we formulate an objective functional ${\mathcal {J}}(u(t))$ as follows:

8 $$\begin{aligned} {\mathcal {J}}(u_{1}(t),u_{2}(t)) = \displaystyle \int _{0}^{T} [A_{1}I_{1} + A_{2}I_{2} + D_{1}u_{1}^{2} + D_{2}u_{2}^{2}]~~~dt, \end{aligned}$$

subject to the system (7), where *T* denotes the final time. This performance specification involves the infectious individuals from both patches and the educational campaign’s cost. $A_{i}$ and $D_{i}$, $i=1,2$ are balancing coefficients (positive) transferring the integral into monetary quantity over a finite period. Note that the control efforts are assumed to be non-linear due to several advantages associated with the non-linear functions of the control. One of the advantages is that a non-linear control lets the Hamiltonian achieve its minimum over the control set at a distinct point. Furthermore, a quadratic structure in control has mathematical advantages. We seek an optimal control pair $(u_{1}^{*},u_{2}^{*}) \in {\mathcal {U}}$ such that

$$\begin{aligned} {\mathcal {J}}(u_{1}^{*},u_{2}^{*}) = \displaystyle \inf _{(u_{1},u_{2}) \in {\mathcal {U}}} {\mathcal {J}}(u_{1},u_{2}), \end{aligned}$$

subject to the state system given by (7), for the admissible set

9 $$\begin{aligned} {\mathcal {U}} = \lbrace (u_{1},u_{2}) \in (L^{\infty } (0,T))^{2} : 0 \le u_{i} \le a_{i} ; ~ a_{i} \in {\mathbb {R}}^{+},~i=1,2 \rbrace . \end{aligned}$$

In the next subsection, we now derive the optimality system.

### Characterization of optimal controls

Utilizing the Pontryagin’s Maximum Principle^63^, the necessary conditions that an optimal control and corresponding states must fulfil are derived. The principle transforms (7) and (8) into a problem of minimizing pointwise a Hamiltonian ${\mathcal {H}},$ regarding $(u_{1}(t),u_{2}(t)):$

$$\begin{aligned} {\mathcal {H}}= & \,\, [A_{1}I_{1} + A_{2}I_{2} + D_{1}u_{1}^{2} + D_{2}u_{2}^{2}]\\&+ \lambda _{1} \bigg [b_{1} - (1-u_{1})\Lambda _{1} S_{1} - \mu _{1}S_{1} + \kappa _{1}R_{1} \bigg ] + \lambda _{2} \bigg [(1-u_{1})\Lambda _{1} S_{1} - (\mu _{1} + \omega _{1} )E_{1}\bigg ] + \lambda _{3} [\omega _{1}E_{1}- (\mu _{1} + \gamma _{1} + v_{1})I_{1}] \\&+ \lambda _{4} [\gamma _{1} I_{1} - (\mu _{1} + \kappa _{1}) R_{1}] + \lambda _{5} [\eta _{1} I_{1} - r_{1}W_{1}] + \lambda _{6} \bigg [b_{2} - (1-u_{2})\Lambda _{2} S_{2} - \mu _{2}S_{2} + \kappa _{2}R_{2} \bigg ] \\&+ \lambda _{7} \bigg [(1-u_{2})\Lambda _{2} S_{2} - (\mu _{2} + \omega _{2} )E_{2}\bigg ] + \lambda _{8} [\omega _{2}E_{2} - (\mu _{2} + \gamma _{2} + v_{2}) I_{2}] + \lambda _{9} [\gamma _{2} I_{2} - (\mu _{2} + \kappa _{2}) R_{2}] + \lambda _{10} [\eta _{2} I_{2} - r_{2}W_{2}], \end{aligned}$$

where $\lambda _{i},~i=1,2,\ldots ,10,$ are the adjoint variables. We now present the adjoint system and control characterization in the following Theorem.

#### Theorem 5

Given an optimal control pair $(u_{1}^{*},u_{2}^{*})$ and corresponding state solutions $S_{i},~E_{i},~I_{i},~R_{i},~W_{i},$ $i=1,2$ of the corresponding state system (7) that minimizes ${\mathcal {J}}(u_{1},u_{2})$ over ${\mathcal {U}},$ there exists adjoint variables (functions), $\lambda _{i}(t),$ for $i = 1,2,\ldots ,10,$ satisfying

10 $$\begin{aligned} \left\{ \begin{array}{llll} \lambda '_{1}(t) &{}=&{} (1-u_{1})\Lambda _{1}(\lambda _{1}-\lambda _{2})+ \mu _{1} \lambda _{1},\\ \lambda '_{2}(t) &{}=&{} \omega _{1}(\lambda _{2}-\lambda _{3}) + \mu _{1} \lambda _{2},\\ \lambda '_{3}(t) &{}=&{}-A_{1}+S_{1}(1-u_{1})\bigg [\dfrac{p^{2}_{11}\beta _{1}}{N_{1}p_{11}+N_{2}p_{21}} +\dfrac{p^{2}_{12}\beta _{2}}{N_{1}p_{12}+N_{2}p_{22}}\bigg ](\lambda _{1}-\lambda _{2})+ \gamma _{1}(\lambda _{3}-\lambda _{4}) - \eta _{1}\lambda _{5}\\ &{}&{}+ S_{2}(1-u_{2})\bigg [\dfrac{p_{11}p_{21}\beta _{1}}{N_{1}p_{11}+N_{2}p_{21}}+\dfrac{p_{12}p_{22} \beta _{2}}{N_{1}p_{12}+N_{2}p_{22}}\bigg ](\lambda _{6}-\lambda _{7})+\lambda _{3}(\mu _{1}+\upsilon _{1}),\\ \lambda '_{4}(t)&{}=&{} \mu _{1}\lambda _{4} + \kappa _{1}(\lambda _{4} - \lambda _{1}),\\ \lambda '_{5}(t) &{}=&{} B_{1}p_{11}\dfrac{S_{1}(1-u_{1})}{K_{1}}(\lambda _{1}-\lambda _{2}) + r_{1} \lambda _{5},\\ \lambda '_{6}(t) &{}=&{} (1-u_{2})\Lambda _{2}(\lambda _{6}-\lambda _{7})+ \mu _{2} \lambda _{6},\\ \lambda '_{7}(t) &{}=&{} \omega _{2}(\lambda _{7}-\lambda _{8}) + \mu _{2} \lambda _{7},\\ \lambda '_{8}(t) &{}=&{}-A_{2}+S_{1}(1-u_{1})\bigg [\dfrac{p_{11}p_{21}\beta _{1}}{N_{1}p_{11}+N_{2}p_{21}}+\dfrac{p_{12}p_{22} \beta _{2}}{N_{1}p_{12}+N_{2}p_{22}}\bigg ](\lambda _{1}-\lambda _{2})+ \gamma _{2}(\lambda _{8}-\lambda _{9}) - \eta _{2}\lambda _{10}\\ &{}&{}+ S_{2}(1-u_{2})\bigg [\dfrac{p^{2}_{21}\beta _{1}}{N_{1}p_{11}+N_{2}p_{21}}+\dfrac{p^{2}_{22}\beta _{2}}{N_{1}p_{12} +N_{2}p_{22}}\bigg ](\lambda _{6}-\lambda _{7})+\lambda _{8}(\mu _{2}+\upsilon _{2}),\\ \lambda '_{9}(t)&{}=&{} \mu _{2}\lambda _{9} + \kappa _{2}(\lambda _{9} - \lambda _{6}),\\ \lambda '_{10}(t) &{}=&{} B_{2}p_{22}\dfrac{S_{2}(1-u_{2})}{K_{2}}(\lambda _{6}-\lambda _{7}) + r_{2} \lambda _{10},\\ \end{array} \right. \end{aligned}$$

with terminal conditions

11 $$\begin{aligned} \lambda _{i}(t) = 0,~~i=1,2,\ldots ,10. \end{aligned}$$

Furthermore, the optimal controls $u_{1}^{*}$ and $u_{2}^{*}$ are represented by

12 $$\begin{aligned} \left\{ \begin{array}{llll} u_{1}^{*}(t) = \max ~\bigg \lbrace 0,~\min ~\bigg (a_{1}, \dfrac{ (\lambda _{2}-\lambda _{1}) \Lambda _{1} S_{1} }{2 D_{1}} \bigg ) \bigg \rbrace , \\ u_{2}^{*}(t) = \max ~\bigg \lbrace 0,~\min ~\bigg ( a_{2}, \dfrac{ (\lambda _{7}-\lambda _{6})\Lambda _{2} S_{2} }{2 D_{2}} \bigg ) \bigg \rbrace . \end{array} \right. \end{aligned}$$

#### Proof

The existence of the optimal control emanates from Corollary 4.1 of^64^ since the integrand of ${\mathcal {J}}$ is a convex function of $(u_{1},u_{2})$ and the state system satisfies the *Lipshitz* property concerning the state variables. From the Pontryagin’s Maximum Principle^63^, the following can be derived

13 $$\begin{aligned} \lambda _{1}' = -\dfrac{\partial {\mathcal {H}}}{\partial S_{1}},~\lambda _{2}' = -\dfrac{\partial {\mathcal {H}}}{\partial E_{1}},~\ldots ,\lambda _{10}' = -\dfrac{\partial {\mathcal {H}}}{\partial W_{2}}, \end{aligned}$$

with $\lambda _{i}(T) = 0$ for $i=1,2,\ldots ,10$ worked out at the optimal controls and accompanying states, which results in the adjoint system (10). ${\mathcal {H}}$ is minimized with respect to the controls at the optimal controls, so we differentiate ${\mathcal {H}}$ concerning $u_{1}$ and $u_{2}$ on the set ${\mathcal {U}},$ respectively, producing the following optimality conditions

14 $$\begin{aligned} \left\{ \begin{array}{llll} \dfrac{\partial {\mathcal {H}}}{\partial u_{1}} = (\lambda _{1} - \lambda _{2})\Lambda _{1} S_{1} + 2D_{1}u_{1} =0, \\ \dfrac{\partial {\mathcal {H}}}{\partial u_{2}} = (\lambda _{6} - \lambda _{7})\Lambda _{2} S_{2} + 2D_{2}u_{2} =0 . \end{array} \right. \end{aligned}$$

Hence, we obtain

15 $$\begin{aligned} \left\{ \begin{array}{llll} u_{1}^{*}(t) = \dfrac{ (\lambda _{2}-\lambda _{1})\Lambda _{1} S_{1} }{2 D_{1}}, \\ u_{2}^{*}(t) = \dfrac{ (\lambda _{7}-\lambda _{6})\Lambda _{2} S_{2} }{2 D_{2}}. \end{array} \right. \end{aligned}$$

Reckoning the bounds on the controls, we obtain the required characterizations. $\square$

#### Remark 1

The uniqueness of the optimal control for a small time (*T*) was obtained, because of the a priori boundedness of the adjoint and state functions and the occurring Lipschitz structure of the ODEs. From the uniqueness of the optimality system, which is composed of Eqs. (7), (10) and (11) with characterization Eq. (12), the uniqueness of the optimal control pair $(u_{1}^{*},u_{2}^{*})$ follows. The limitation on the length of the time gap is to guarantee the uniqueness of the optimality system, the minute size in the length of time is due to the opposite time orientations of Eqs. (7), (10) and (11); the adjoint problem has final values and the state problem has initial values. The limitation is prevalent in control problems (see^65–72^)

## Numerical simulations

Utilizing the parameters in Table 1 and the forward-backward sweep method (implemented in Matlab), we work out the numerical solutions to the optimality system consisting of adjoint equation (13), state equation (7), corresponding initial/final conditions and control characterizations. The algorithm is initialized with a guess for the optimal controls, and the state variables are then worked forward in time using the Runge Kutta fourth-order method. Employing the backward Runge Kutta of the fourth-order, the initial control guess, and the state variables are used to solve the adjoint equations (13) backward in time with given final conditions. The controls $u_{1}$ and $u_{2}$ are then updated and used to solve the state and the adjoint system. When the current state, adjoint, and control values converge sufficiently^73^, this iterative process terminates.

In this section we utilize numerical simulations to support the analytic results previously established, and to provide examples about the dynamics of COVID-19. We use the following initial conditions:

$$\begin{aligned} S_{i}(0) = 0.7,~ E_{i}(0) = 0.25,~ I_{i}(0) = 0.05,~ R_{i}(0) = 0.0. \end{aligned}$$

We made use of the Parameters in Table 1, that have been extracted from the work done in^74^, which reflect the historic lineage. Our main aim is to explore the effects of optimal preventive measures on the transmission dynamics of COVID-19 with different coupling intensities under the following cases:

- Case 1 : Less efficient preventative measures in the high risk patch, that is patch 1, and efficient preventative measures in the low risk patch, that is patch 2. Thus, $u_{1} =0.45$ and $u_{2}=0.80.$
- Case 2 : Efficient preventative measures in the high risk patch, that is patch 1, and efficient preventative measures in the low risk patch, that is patch 2. Thus, $u_{1} =0.80$ and $u_{2}=0.80.$

**Figure 2:**
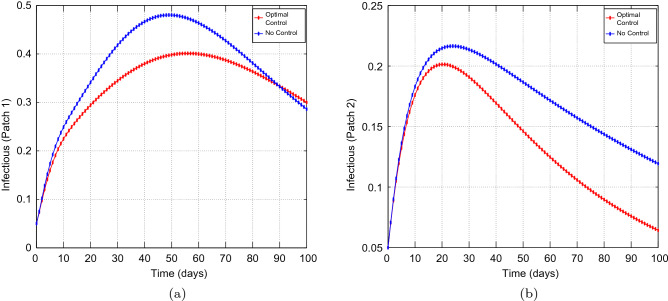
Simulation outcomes for the presented two patch COVID-19 mathematical model for **case 1** under symmetric weak coupling (**a**) the number of infected humans in patch 1 (**b**) the number of infected humans in patch 2. In both figures, the red curves and blue curves are for the infected population, with optimal control and no optimal control respectively.

**Figure 3:**
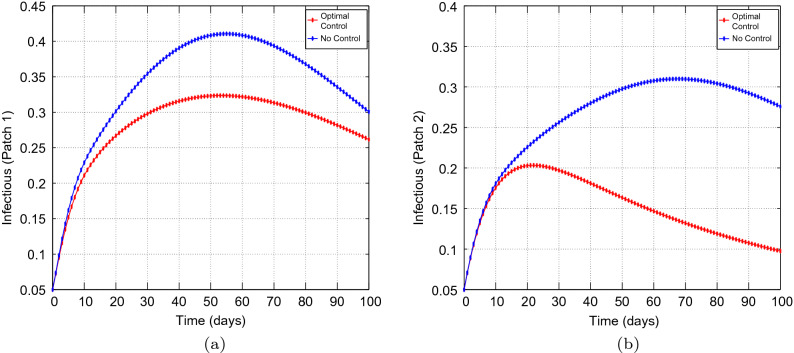
Simulation outcomes for the presented two patch COVID-19 mathematical model for **case 1** under symmetric strong coupling (**a**) the number of infected humans in patch 1 (**b**) the number of infected humans in patch 2. In both figures, the red curves and blue curves are for the infected population, with optimal control and no optimal control respectively.

**Figure 4:**
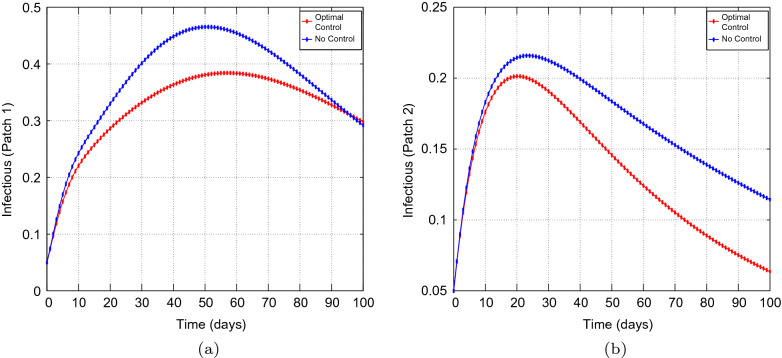
Simulation outcomes of the presented two patch COVID-19 mathematical model for **case 1** under asymmetric weak coupling (**a**) the number of infected humans in patch 1 (**b**) the number of infected humans in patch 2. In both figures, the red curves and blue curves represent the infected population, with optimal control and no optimal control respectively.

**Figure 5:**
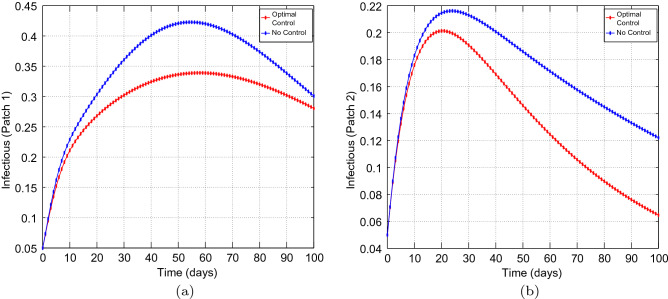
Simulation outcomes for the presented two patch COVID-19 mathematical model for **case 1** under asymmetric strong coupling (**a**) the number of infected humans in patch 1 (**b**) the number of infected humans in patch 2. In both figures, the red curves and blue curves are for the infected population, with optimal control and no optimal control respectively.

Figures 1, 2, 3, 4 and 5 depicts the impact of coupling intensities on the two patches under Case 1, over a period of 100 days. Particularly, Figs. 1, 2, 3, 4 and 5 illustrates the number of infected individuals per patch, with and without optimal control under weak symmetric coupling, strong symmetric coupling, weak asymmetric coupling and strong asymmetric coupling, respectively. Whenever the coupling is weak, we can see that the optimal control policy does not have a significant impact and not effective for the whole 100 days as compared to patch 2 in which the infections reduce with time. However, when the coupling intensity is strong, the number of the infected individuals in both patches reduce with time but with more impact being observed in patch 2 where there are effective control measures.

**Figure 6:**
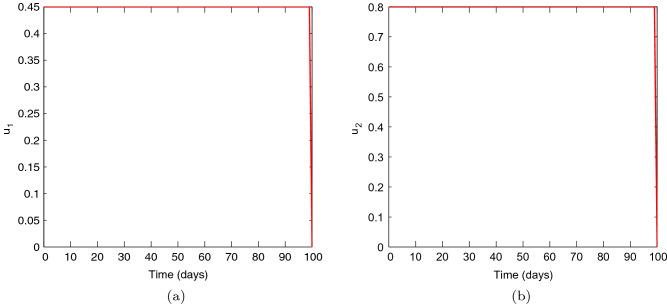
The control profile for case 1: (**a**) Patch 1; and (**b**) Patch 2.

Figure 6 represents the controls $u_{1}$ and $u_{2}.$ The patch 1 control $u_{1}$ is at the upper bound $b_{1}$ for approximately 100 days and has a sharp drop until it reaches the lower bound. The patch 2 control $u_{2}$ is also at the upper bound for approximately 100 days and has a sharp drop until it reaches the lower bound. These results suggest that more effort can be devoted to both controls.

**Figure 7:**
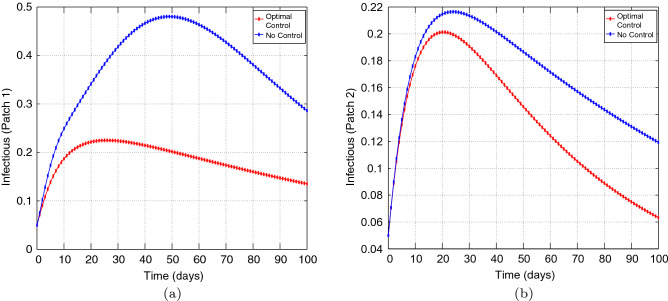
Simulation outcomes for the presented two patch COVID-19 mathematical model for **case 2** under symmetric weak coupling (**a**) the number of infected humans in patch 1 (**b**) the number of infected humans in patch 2. In both figures, the red curves and blue curves are for the infected population, with optimal control and no optimal control respectively.

**Figure 8:**
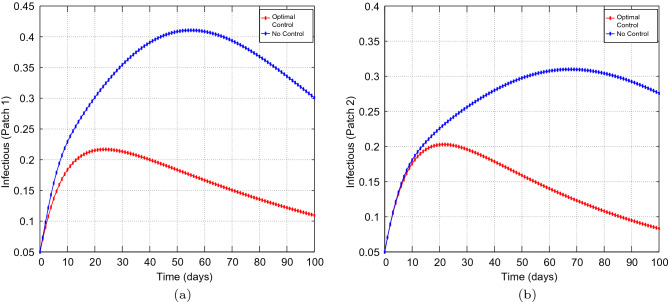
Simulation outcomes of the presented two patch COVID-19 mathematical model for **case 2** under symmetric strong coupling (**a**) the number of infected humans in patch 1 (**b**) the number of infected humans in patch 2. In both figures, the red curves and blue curves represent the infected population, with optimal control and no optimal control respectively.

**Figure 9:**
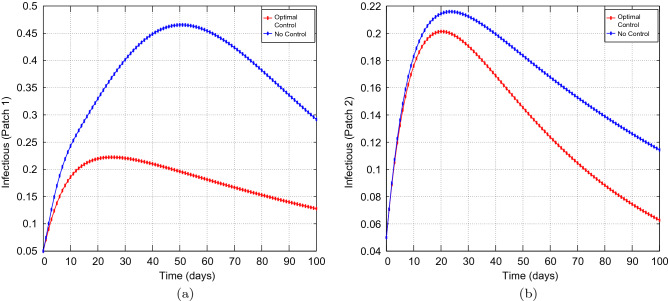
Simulation outcomes for the presented two patch COVID-19 mathematical model for **case 2** under asymmetric weak coupling (**a**) the number of infected humans in patch 1 (**b**) the number of infected humans in patch 2. In both figures, the red curves and blue curves are for the infected population, with optimal control and no optimal control respectively.

**Figure 10:**
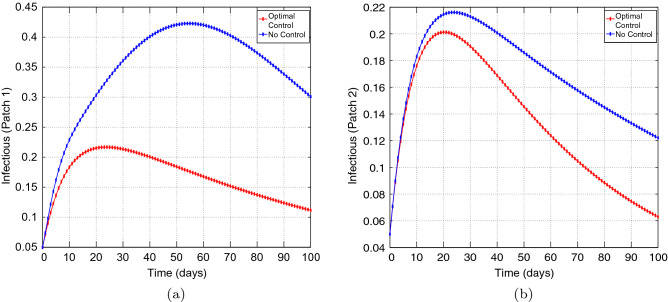
Simulation outcomes for the presented two patch COVID-19 mathematical model for **case 2** under asymmetric strong coupling (**a**) the number of infected humans in patch 1 (**b**) the number of infected humans in patch 2. In both figures, the red curves and blue curves are for the infected population, with optimal control and no optimal control respectively.

Figures 6, 7, 8, 9 and 10 illustrates the impact of effective control measures for both patches, which represents case 2. We can see that the total number of of the infected individuals for both patches decreases as a result of the optimal policy.

**Figure 11:**
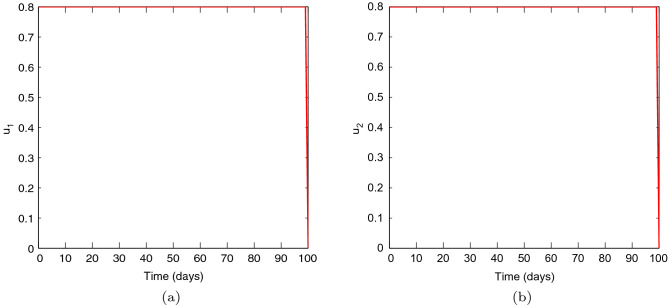
The control profile for case 1: (**a**) Patch 1; and (**b**) Patch 2.

Figure 11 depicts the impact of the controls $u_{1}$ and $u_{2}.$ The patch 1 control $u_{1}$ is at the upper bound $b_{1}$ for approximately 100 days and has a sharp drop until it reaches the lower bound. The patch 2 control $u_{2}$ is also at the upper bound for approximately 100 days and has a sharp drop until it reaches the lower bound. We conclude that both controls are important in controlling COVID-19.

## Cost-effectiveness analysis

To show to be just with the costs associated with the control strategies, the educational campaigns as preventive measures, the incorporated benefits are normally evaluated using the cost-effectiveness analysis. We shall borrow the same ideas from the cost-effectiveness analysis, and in attaining this, we will have to compare the differences between the health outcomes and the costs of the respective coupling intensities. Thus, we will make use of two approaches, namely the average cost-effectiveness ratio (ACER) and the infection averted ratio (IAR).

Before we investigate the IAR and the ACER, we shall first determine the infections averted and the costs associated with each coupling intensity under our control strategy.

**Table 3:** The total number of infections averted over a 100 day period.

Cases	Case 1	Case 2	${\mathcal {{{R}}}}_{0}$
Weak symmetric coupling	7.825620	22.91336	3.96
Strong symmetric coupling	17.09808	29.12621	2.82
Weak asymmetric coupling	7.923208	22.42164	3.84
Strong asymmetric coupling	8.845054	21.45402	3.79

The number of infections averted is computed as the difference between the total infectious individuals without control and the total infectious individuals with the control. In Table 3, we notice that most infections are averted under strong symmetric coupling. It is worth noting that for both cases, case 1 and case 2, more infections are averted under symmetric coupling. This clearly portrays that increased mobility between the two risk communities reduces the overall epidemic size.

**Table 4:** The total cost with respect to the control policy, over a 100 day period.

Cases	Case 1	Case 2	${\mathcal {{{R}}}}_{0}$
Weak symmetric coupling	4.70407 $\times ~ 10^{5}$	3.19530 $\times ~ 10^{5}$	3.96
Strong symmetric coupling	4.34942 $\times ~ 10^{5}$	3.14661 $\times ~ 10^{5}$	2.82
Weak asymmetric coupling	4.59452 $\times ~ 10^{5}$	3.14468 $\times ~ 10^{5}$	3.84
Strong asymmetric coupling	4.30111 $\times ~ 10^{5}$	3.04021 $\times ~ 10^{5}$	3.79

Table 4 illustrates that, employing the control policy in trying to curtail the spread of COVID-19 it is more expensive under the weak symmetric coupling. Thus, if those within the low risk patch and those in the high risk patch stay within their respective patches, the disease becomes expensive to control. We observe that the lowest cost of controlling the disease is under strong asymmetric coupling, that is when more people from the low risk move to the high risk patch.

From Tables 3 and 4, an interesting result is discovered, we noticed that more infections are averted under strong symmetric coupling yet to implement our strategies, the cheapest way would be under strong asymmetric coupling. Hence, to further understand this phenomenon we then employ the IAR and the ACER.

### Infection averted ratio (IAR)

The infection averted ratio is defined as

16 $$\begin{aligned} \left\{ \text{ IAR } = \dfrac{\text{ Number } \text{ of } \text{ infections } \text{ averted }}{\text{ Number } \text{ of } \text{ recovered }}. \right. \end{aligned}$$

The number of infections averted is defined as the difference between the total infectious individuals without the control and the total infectious individuals with the control. The most effective strategy is the one with the highest IAR.

### Average cost-effectiveness ratio (ACER)

The average cost-effectiveness ratio deals with a single intervention, evaluating it against the zero intervention baseline option. It is defined as

17 $$\begin{aligned} \left\{ \text{ ACER } = \dfrac{\text{ Total } \text{ cost } \text{ produced } \text{ by } \text{ the } \text{ intervention }}{\text{ Total } \text{ number } \text{ of } \text{ infection } \text{ averted }}. \right. \end{aligned}$$

The most cost-effective strategy, is the one with the lowest ACER.

After doing the necessary computations and also making use of Matlab programming in calculating the respective values, we present the following figures. *A* representing weak symmetric coupling $({\mathcal {R}}_{0} = 3.96)$, *B* representing strong symmetric coupling $({\mathcal {R}}_{0} = 2.82)$, *C* representing weak asymmetric coupling $({\mathcal {R}}_{0} = 3.84)$ and *D* representing strong asymmetric coupling $({\mathcal {R}}_{0} = 3.79)$.

**Figure 12:**
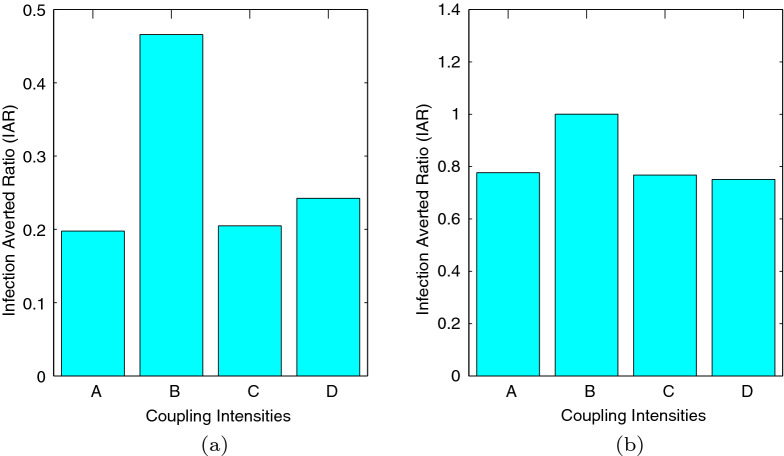
Graphs of (**a**) showing the infection averted ratio (IAR) for case 1 and (**b**) showing the infection averted ratio (IAR) for case 2.

From Fig. 12, we see that the infection averted ratio is highest under strong symmetric coupling. Thus, implementing our control policy under strong symmetric coupling would be the most ideal, since more lives would be saved. Practically, use of *cordon sanitaires* or community lockdowns are not always the best methods of reducing pandemics. Furthermore, the IAR would be highest under case 2 when both control strategies would be efficient at $80\%$. An interesting result is noted in that the IAR for the strong asymmetric coupling under case 1, is almost equal to the IAR for weak symmetric coupling, weak asymmetric coupling and strong strong asymmetric coupling under case 2. Thus, implementing educational campaigns bears more fruits under strong symmetric coupling.

**Figure 13:**
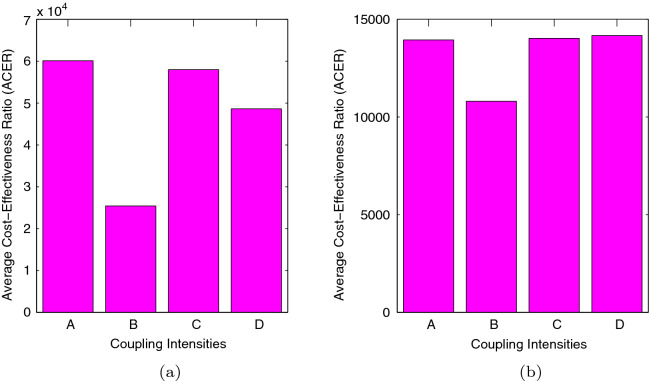
Graphs of (**a**) showing the average cost-effectiveness ratio (ACER) for case 1 and (**b**) showing the average cost effectiveness ratio (ACER) for case 2.

Figure 13 depicts ACER for both case 1 and case 2. On comparing case 1 and case 2, we can see that we have our lowest ACER under case 1, when both intervention strategies would be implemented with high efficiency. Implementing our strategy would be the most ideal under strong symmetric coupling, for both cases.

## Discussion

We have proposed and studied a model to understand the impact of short-term human movement on transmission and the control of COVID-19 within a heterogeneous population. The suggested mathematical model consists of two patches and human movement modeled through a Lagrangian approach. This study applies within and between countries where two communities differ by risk (high risk and low risk) and are under strict lockdown. Furthermore, it applies to the country’s borders, where two countries differ economically, such as in the case of Zimbabwe and South Africa. The heterogeneity in human movement contacts may significantly contribute to the transmission and control of COVID-19.

We computed and analyzed the threshold quantity known as the reproduction number ${\mathcal {R}}_{0}$, for our proposed mathematical model. We noted that the reproduction number is a function of various factors such as the proportion of the time that individuals of each patch spend in their patch and the other patch, rates of transmission, life expectancy of the pathogens in the environment, death due to COVID-19, natural death rate, etc. We also noted that the reproduction number ${\mathcal {R}}_{0}$, depends on the characteristics of both patches. But, without human mobility we realized that each patch has its own reproduction number, ${\mathcal {R}}_{01}$ and ${\mathcal {R}}_{02}$ for patch one and patch two, respectively. It is worth stating that each patch depends on the characteristics of each respective patch. Numerically, we managed to show that in the absence of human mobility ${\mathcal {R}}_{01} =1.20690$ and ${\mathcal {R}}_{02} =0.42138$, which means that COVID-19 dies out in the low-risk patch and persists in the high-risk patch. We noted that in the presence of human mobility, the reproduction number would always be greater than 2, meaning that mobility increases the spread of the disease in the community. Hence, lockdowns are justified in reducing COVID-19 prevalence. We noted that the reproduction number would be the highest when the mobility pattern is symmetric and the coupling intensity is weak, at ${\mathcal {R}}_{0}=1$. We then employed some analytic methods to show that when ${\mathcal {R}}_{0} \le 1$, COVID-19 dies out within the community. When ${\mathcal {R}}_{0} > 1$, an endemic equilibrium point that is unique exists, and the disease persists.

We then used the optimal control theory to determine the optimal strategy for reducing COVID-19. The strategy implemented was educational campaigns within each respective patch. We investigated two cases that mimic COVID-19 strategies in developing countries. Case 1, it represents the environment in which there would be less efficient measures in the high risk patch (taking $u_{1} = 0.45$) and efficient preventive measures in patch (taking $u_{1} = 0.80$). This is the scenario in which there is insufficient information in the real world and no means of reaching out efficiently to those at high risk of contracting COVID-19. Case 2, we have efficient preventive measures in both patches, that is we set $u_{1} = 0.80$ and $u_{2} = 0.80$ for both cases. Under Case 1, we noted that the controls are more effective under weak symmetric coupling. Furthermore, under Case 2, we also noted that the controls are effective under strong symmetric coupling. The numerical results provide important evidence that having effective controls on both patches is the best way forward. Our results show that human mobility plays a vital role in directing the long-term dynamics of COVID-19, which later impacts the design of its optimal control strategies.

We employed the cost-effectiveness analysis to understand the costs associated with the control strategies. More infections are averted under strong symmetric coupling, which its low reproduction number may justify. Additionally, for both cases, we noted that it is expensive to control COVID-19 under weak symmetric coupling, the cheapest being strong asymmetric coupling. To fully understand the costs associated with our control strategies, we calculated the infection averted ratio (IAR) and the average cost-effectiveness ratio (ACER). The highest AIR was attained under strong symmetric coupling for both cases. The lowest ACER was observed under strong symmetric coupling. Thus, it is beneficial and cost-effective for both cases to carry out the controls under strong symmetric coupling to curtail the disease faster. Lastly, the best way would be to control the disease without movement.

### Limitations

Our study consists of some limitations. Limited data exist on COVID-19 regarding short-term dispersal, particularly not much mathematical modeling has been done concerning short-term dispersal. Therefore, some of our numerical estimates remain uncertain. Thus, we had to utilize data published in the literature, to base our numerical results. More data sets and experimental studies are needed to include more natural biological processes in the models of short-term human movements. Our constant $K_{i}$, which represents the number or quantity of pathogens present during human interaction, is independent of time. Lastly, we did not include the age structure, which is also a variable in the mode of transmission of COVID-19. It was shown that the susceptibility profile among children is much less than the elderly population, but for our main aim, this was not very essential.

### Future perspectives

However, just like any other model, we cannot say the model is complete. Hence the following improvements can be included in the model in the future. It is well known that pre-symptomatic individuals play an essential role in the spread of COVID-19. Hence the pre symptomatic, symptomatic and asymptomatic individuals can be treated as separate classes. The paper is theoretical and does not include empirical data. It would be essential to calibrate the model with real empirical data. Lastly, the manuscript investigated the infected averted ratio (IAR) and the average cost-effectiveness ratio (ACER). It would be necessary also to include the incremental cost-effectiveness ratio (ICER). The ICER is the additional cost per additional health outcome and we assume that the costs of the various control interventions are directly proportional to the number of controls deployed.

## Supplementary Information

Supplementary Information.

## Data Availability

The data used to support the findings of this study are included within the article and cited accordingly.

## References

[1] Cases, Data, and Surveillance. Centers for Disease Control and Prevention. 11 February 2020. Retrieved 11 February 2021.

[2] World Health Organization (WHO) Q and A on coronaviruses (COVID-19). https://www.who.int/emergencies/diseases/novel-coronavirus-2019/question-and-answers-hub/q-a-detail/q-a-coronaviruses (Accessed July 16 2021).

[3] CDC, Centers for disease control and prevention, Symptoms of COVID-19. https://www.cdc.gov/coronavirus/2019-ncov/symptoms-testing/symptoms.html/ (2021).

[4] Chen, T., Rui,J., Wang, Q., Zhao, Z., Cui, J. & Yin, L. A mathematical model for simulating the phase-based transmissibility of a novel coronavirus. *Infect. Dis. Poverty* **9**(1) (2020). 10.1186/s40249-020-00640-3 PMC7047374 32111262

[5] He S, Tang S, Rong L (2020). A discrete stochastic model of the COVID-19 outbreak: Forecast and control. Math. Biosci. Eng..

[6] Bai Y, Yao L, Wei T, Tian F, Jin DY, Chen L, Wang M (2020). Presumed asymptomatic carrier transmission of COVID-19. JAMA.

[7] Rothe C, Schunk M, Sothmann P, Bretzel G, Froeschl G, Wallrauch C, Zimmer T, Thiel V, Janke C, Guggemos W, Seilmaier M, Drosten C, Vollmar P, Zwirglmaier K, Zange S, Wolfel R, Hoelscher M (2020). Transmission of 2019-nCoV infection from an asymptomatic contact in Germany. N. Engl. J. Med..

[8] Zou L, Ruan F, Huang M, Hong Z, Yu J, Kang M, Song Y, Xia J, Guo Q, Song T, He J, Yen HL, Peiris M, Wu J (2020). SARS-CoV-2 viral load in upper respiratory specimens of infected patients. N. Engl. J. Med..

[9] Meyerowitz, E. A., Richterman, A., Gandhi, R. T. & Sax, P. E. Transmission of SARS-CoV-2: A review of viral, host, and environmental factors. *Ann. Int. Med.* (2020). 10.7326/M20-5008 PMC7505025 32941052

[10] Kampf, G., *et al.* Potential sources, modes of transmission and effectiveness of prevention measures against SARS-CoV-2. *J. Hosp. Infect.* (2020). 10.1016/j.jhin.2020.09.022 PMC7500278 32956786

[11] Pastorino B, Touret F, Gilles M, de Lamballerie X, Charrel RN (2020). Prolonged Infectivity of SARSCoV-2 in Fomites. Emerg. Infect. Dis..

[12] Worldometer. https://www.worldometers.info/coronavirus/. (Accessed 26 July 2021).

[13] Spicer AJ, Jalkanen S (2021). Why haven’t we found an effective treatment for COVID-19?. Front. Immunol..

[14] Pérez-Alós L, Armenteros JJA, Madsen JR (2022). Modeling of waning immunity after SARS-CoV-2 vaccination and influencing factors. Nat. Commun..

[15] Adepoju, P. Africa prepares for endemic COVID-19, The pandemic is far from over in Africa, but there is also a funding gap in preparing for endemic COVID-19, which will require long-term investment in healthcare infrastructure. 10.1038/d41591-022-00040-0.

[16] IOM, UN Migration, Migration for Development: Within and Beyond Frontiers, 978-92-9068-310-X, IOM Publications platform.

[17] https://www.statista.com/statistics/1120999/gdp-of-african-countries-by-country/.

[18] Vaccine tourism: South Africans cross border to Zimbabwe for Covid-19 jab, https://www.sowetanlive.co.za/news/south-africa/2021-05-08-vaccine-tourism-south-africans-cross-border-to-zimbabwe-for-covid-19-jab/ (2021).

[19] eNCA, COVID-19 vaccine, South Africans getting a jab in Zimbabwe. https://www.youtube.com/watch?v=Jnfur4u3LR8.

[20] Perrings C, Espinoza B (2021). Mobility restrictions and the control of COVID-19. Ecol. Econ. Soc. INSEE J..

[21] https://hungrycities.net/planning-responses-to-covid-19-in-zimbabwean-cities/.

[22] https://www.worldbank.org/en/country/zimbabwe/publication/zimbabwe-economic-update-covid-19-further-complicates-zimbabwe-s-economic-and-social-conditions.

[23] Zimbabwe Humanitarian Situation Report 2021, Zimbabwe COVID-19 Daily Sitrep. 30/06/2021, Ministry of Health and Child Care

[24] The impact of COVID 19 on socio-economic rights in Zimbabwe, Zimbabwe Peace Project, http://www.zimpeaceproject.com.

[25] Bedford J, Enria D, Giesecke J, Heymann D, Ihekweazu C, Kobinger G, Lane H, Memish Z, Oh M, Sall A, Schuchat A, Ungchusak K, Wieler L (2020). Covid-19: Towards controlling of a pandemic. Lancet.

[26] Yavuz M, Cosar F, Gunay, Ozdemir FN (2021). A new mathematical modeling of the COVID-19 pandemic including the vaccination campaign. World J. Model. Simul..

[27] Ghinai I, McPherson TD, Hunter JC, Kirking HL, Christiansen D, Joshi K, Rubin R, Morales-Estrada S, Black SR, Pacilli M, Fricchione MJ, Chugh RK, Walblay KA, Ahmed NS, Stoecker WC, Hasan NF, Burdsall DP, Reese HE, Wallace M, Wang C, Moeller D, Korpics J, Novosad SA, Benowitz I, Jacobs MW, Dasari VS, Patel MT, Kauerauf J, Charles EM, Ezike NO, Chu V, Midgley CM, Rolfes MA, Gerber SI, Lu X, Lindstrom S, Verani JR, Layden J.E. Illinois (2020). COVID-19 Investigation Team. First known person-to-person transmission of severe acute respiratory syndrome coronavirus 2 (SARS-CoV-2) in the USA. Lancet.

[28] Ahmed I, Modu GU, Yusuf A, Kumam P, Yusuf I (2021). A mathematical model of Coronavirus Disease (COVID-19) containing asymptomatic and symptomatic classes. Res. Phys..

[29] Adiga A, Dubhashi D, Lewis B, Marathe M, Venkatramanan S, Vullikanti A (2020). Mathematical models for COVID-19 pandemic: A comparative analysis. J. Indian Inst. Sci..

[30] Kyrychko YN, Blyuss KB, Brovchenko I (2020). Mathematical modelling of the dynamics and containment of COVID-19 in Ukraine. Sci. Rep..

[31] Zeb A, Alzahrani E, Erturk VS, Zaman G (2020). Mathematical model for coronavirus disease 2019 (COVID-19) containing isolation class. Biomed. Res. Int..

[32] Mugisha JYT, Ssebuliba J, Nakakawa JN, Kikawa CR, Ssematimba A (2021). Mathematical modeling of COVID-19 transmission dynamics in Uganda: Implications of complacency and early easing of lockdown. PLoS ONE.

[33] Mwalili S, Kimathi M, Ojiambo V, Gathungu D, Mbogo R (2020). SEIR model for COVID-19 dynamics incorporating the environment and social distancing. BMC. Res. Notes.

[34] Garba SM, Lubuma JMS, Tsanou B (2020). Modeling the transmission dynamics of the COVID-19 Pandemic in South Africa. Math. Biosci..

[35] Aldila D, Ndii MZ, Samiadji BM (2020). Optimal control on COVID-19 eradication program in Indonesia under the effect of community awareness. Math. Biosci. Eng..

[36] Liu, H. & Tian, X. Data-driven optimal control of a seir model for COVID-19. *Commun. Pure Appl. Anal.*10.3934/cpaa.2021093.

[37] Silva CJ, Cruz C, Torres DFM, Munuzuri PA, Carballosa A, Area I, Nieto JJ, Fonseca-Pinto R, Passadouro R, Soares dos Santos E, Abreu W, Mira J (2021). Optimal control of the COVID-19 pandemic: Controlled sanitary deconnement in Portugal. Sci. Rep..

[38] Gatyeni, S. P., Chukwu, C.W., Chirove, F., Fatmawati & Nyabadza, F. Application of Optimal Control to the Dynamics of COVID-19 Disease in South Africa. medRxiv. 10.1101/2020.08.10.20172049 (2020). 10.1016/j.sciaf.2022.e01268 PMC9245336 35791321

[39] Arruda, E. F., Pastore, D. H., Dias, C. M. & Das, S.S. Modelling and optimal control of multi strain epidemics, with application to COVID-19. arXiv:2101.08137v1. 10.1371/journal.pone.0257512 PMC8445490 34529745

[40] Madubueze, C. E., Dachollom, S. & Onwubuya, I.O. Controlling the Spread of COVID-19: Optimal Control Analysis. *Comput. Math. Methods Med.* **2020** Article ID 6862516 (2020). 10.1155/2020/6862516 PMC7499329 32963585

[41] Zakary, O. Bidah, S. Rachik, M. & Ferjouchia, H. Mathematical model to estimate and predict the COVID-19 infections in Morocco: Optimal control strategy. *J. Appl. Math.* **2020** Article ID 9813926 (2020).

[42] Richard Q, Alizon S, Choisy M, Sofonea MT, Djidjou-Demasse R (2021). Age-structured nonpharmaceutical interventions for optimal control of COVID-19 epidemic. PLoS Comput. Biol..

[43] Kouidere A, Youssoua L, Ferjouchia H, Balatif O, Rachik M (2021). Optimal Control of Mathematical modeling of the spread of the COVID-19 pandemic with highlighting the negative impact of quarantine on diabetics people with Cost-effectiveness. Chaos Solit. Fractals..

[44] Tilahun, G. T. & Alemneh, H. T. Mathematical modeling and optimal control analysis of COVID-19 in Ethiopia. *J. Interdiscip. Math.*10.1080/09720502.2021.1874086 (2021).

[45] Olaniyi S, Obabiyi OS, Okosun KO, Oladipo AT, Adewale SO (2020). Mathematical modelling and optimal cost-effective control of COVID-19 transmission dynamics Eur. Phys. J. Plus..

[46] Malinzi J, Ouifki R, Eladdadi A, Torres DFM, White KAJ (2018). Enhancement of chemotherapy using oncolytic virotherapy: Mathematical and optimal control analysis. Math. Biosci. Eng..

[47] Sharomi O, Malik T (2017). Optimal control in epidemiology. Ann. Oper. Res..

[48] Area, I., Ndaïrou, F., Nieto, J. J., Silva, C. J. & Torres, D. F. M. Ebola model and optimal control with vaccination constraints. *J. Ind. Manage. Optim.* (2017).

[49] Khan MA, Shah SW, Ullah S, Gómez-Aguilar JF (2019). A dynamical model of asymptomatic carrier Zika virus with optimal control strategies. Nonlinear Anal. RealWorld Appl..

[50] Silva CJ, Torres DFM (2018). Modeling and optimal control of HIV/AIDS prevention through prep. Discrete Contin. Dyn. Syst..

[51] Espinoza B, Castillo-Chavez C, Perrings C (2020). Mobility restrictions for the control of epidemics: When do they work?. PLoS ONE.

[52] Espinoza, B., Moreno, V., Bichara, D. & Castillo-Chavez, C. Assessing the efficiency of movement restriction as a control strategy of Ebola. In: Mathematical and Statistical Modeling for Emerging and Re-emerging Infectious Diseases (eds. Chowell, G. & Hyman, J.) (Springer, 2016). 10.1007/978-3-319-40413-4-9.

[53] Bichara D, Iggidr A (2018). Multi-patch and multi-group epidemic models: A new framework. J. Math. Biol..

[54] Bichara D, Castillo-Chavez C (2016). Vector-borne diseases models with residence times: A Lagrangian perspective. Math. Biosci..

[55] Bichara, D., Kang, Y., Castillo-Chavez, C., Horan, R. & Perrings, C. *Bull. Math. Biol.* **77**(11), 2004–2034 (2015). 10.1007/s11538-015-0113-5 PMC4749480 26489419

[56] Agusto, F., Goldberg, A., Ortega, O., Ponce, J., Zaytseva, S., Sindi, S. & Blower, S. How do interventions impact malaria dynamics between neighbouring countries? A case study with Botswana and Zimbabwe, In book: Using Mathematics to Understand Biological Complexity (pp. 83–109, 2021).

[57] Kim JE, Lee H, Lee CH, Lee S (2017). Assessment of optimal strategies in a two-patch dengue transmission model with seasonality. PLoS ONE.

[58] Castillo Chavez, C. Song, B. & Zhangi, J. An epidemic model with virtual mass transportation: The case of smallpox, Bioterrorism. (Mathematical Modeling Applications in Homeland Security, vol 28, 2003).

[59] Van den Driessche P, Watmough J (2002). Reproduction numbers and sub-threshold endemic equilibria for compartmental models of disease transmission. Math. Biosci..

[60] McCluskey CC (2006). Lyapunov functions for tuberculosis models with fast and slow progression. Math. Biosci. Eng..

[61] Shuai Z, Heesterbeek JAP, van den Driessche P (2013). Extending the type reproduction number to infectious disease control targeting contact between types. J. Math. Biol..

[62] Mhlanga A, Mupedza TV, Mazikana TM (2022). Optimal control and cost-effective analysis of a scabies model with direct and indirect transmissions. J. Biol. Syst..

[63] Pontryagin LS, Boltyanskii VT, Gamkrelidze RV, Mishchevko EF (1985). The Mathematical Theory of Optimal Processes.

[64] Fleming WH, Rishel RW (1975). Deterministic and Stochastic Optimal Control.

[65] Agusto FB (2013). Optimal isolation control strategies and cost-effectiveness analysis of a two-strain avian influenza model. Biosyst. Eng..

[66] Agusto FB, Lenhart S (2013). Optimal control of the spread of malaria superinfectivity. J. Biol. Syst..

[67] Agusto FB, Marcus N, Okosun KO (2012). Application of optimal control to the epidemiology of malaria. Electron. J. Differ. Equ..

[68] Joshi HR (2002). Optimal control of an HIV immunology model. Optim Control Appl Methods..

[69] Kirschner D, Lenhart S, Serbin S (1997). Optimal control of the chemotherapy of HIV. J. Math. Biol..

[70] Mhlanga, A. Dynamics of HSV-2 in the presence of optimal counseling and education among prisoners. *Discrete Dyn Nat Soc.* Article ID 9916427 (2021).

[71] Mhlanga, A. Bhunu, C.P. Mushayabasa, S. A Computational Study of HSV-2 with poor treatment adherence. *Abstr. Appl. Anal.* Article ID 850670, (2015).

[72] Mhlanga A (2018). A theoretical model for the transmission dynamics of HIV/HSV-2 co-infection in the presence of poor HSV-2 treatment adherence. Appl. Math. Nonlinear Sci..

[73] Lenhart S, Workman JT (2007). Optimal Control Applied to Biological Models.

[74] Mwalili, S.M., Kimathi, M., Ojiambo, V., Gathungu, D.K. & Mbongo, R.W. SEIR model for COVID-19 dynamics incorporating the environment and social distancing. Research Square. 10.1186/s13104-020-05192-1 PMC7376536 32703315

