# Supplementary material for: A patchy theoretical model for the transmission dynamics of SARS-Cov-2 with optimal control
Source: Sci Rep. 2022 Oct 25;12:17840. doi: 10.1038/s41598-022-21553-1 (PMC9592884; doi:10.1038/s41598-022-21553-1)
Supplement: Supplementary file 1 — Supplementary Information. [file 41598_2022_21553_MOESM1_ESM.pdf]

Supplemetary Material

♣ A. Mhlanga, ♢ T.V. Mupedza *
♣ *Department of Epidemiology and Biostatistics, Indiana University School of Public Health, Bloomington, IN, USA*
♢ *Department of Mathematics, University of Zimbabwe, Box MP 167 Mount Pleasant, Harare, Zimbabwe*
* Corresponding author: Email: *tvmupedza@science.uz.ac.zw*

Appendix A

Proof of Theorem 2.

Proof. Let $\Theta(t) = (E_i(t), I_i(t), W_i(t))^T$. Since from (1)

$$\begin{cases} E_i' = B_i p_{ii} S_i \dfrac{W_i}{N_i} + \displaystyle\sum_{j=1}^{2} \beta_j p_{ij} S_i \dfrac{\sum_{k=1}^{2} p_{kj} I_k}{\sum_{k=1}^{2} p_{kj} N_k} - (\mu_i + \omega_i)E_i, \\ I_i' = \omega_i E_i - (\gamma_i + \mu_i + v_i)I_i, \\ W' = \eta_i I_i - r_i W_i, \end{cases} \tag{1}$$

it follows that

$$\dot{\Theta}(t) \leq (F - V)\Theta, \tag{2}$$

with F and V as presented in equation (4). It is worth mentioning that the matrices F and V^{-1} are non-negative. Employing the Perron-Frobenius Theorem [1], the non-negative matrix $V^{-1}F$ has a non-negative left eigenvector u concerning the eigenvalue $\mathcal{R}_0 = \rho(V^{-1}F) = \rho(FV^{-1})$; that is, $u^T V^{-1} F = \mathcal{R}_0 u^T$. Inspired by [2], we utilize a Lyapunov function

$$\mathcal{Y}(t) = u^T V^{-1} \Theta. \tag{3}$$

Differentiating $\mathcal{Y}(t)$ along solutions of (1), we get

$$\begin{aligned} \dot{\mathcal{Y}}(t) &= u^T V^{-1} \dot{\Theta} \\ &\leq u^T V^{-1}(F - V)\Theta \\ &= (\mathcal{R}_0 - 1)u^T \Theta \leq 0 \quad \text{if } \mathcal{R}_0 \leq 1. \end{aligned} \tag{4}$$

It can be found that the largest invariant subset of $\mathcal{G}$ where $\dot{\mathcal{Y}} = 0$ is the singleton $\{\mathcal{E}^0\}$. Accordingly, by the invariance principle of LaSalle [3], $\mathcal{E}^0$ is globally asymptotically stable in $\mathcal{G}$ when $\mathcal{R}_0 \leq 1$.

If $\mathcal{R}_0 > 1$, subsequently by continuity, $\dot{\mathcal{Y}} > 0$ in the neighbourhood of $\mathcal{E}^0$ in the interior of $\mathcal{G}$. Solutions sufficiently close to $\mathcal{E}^0$ in the interior of $\mathcal{G}$, retreat from the DFE meaning that the DFE is unstable. Utilizing a result from Thieme on uniform persistence [4] and an argument as in the proof of Proposition 3.3 of [5], it can be established that when $\mathcal{R}_0 > 1$, the uniform persistence of system (1) follows from the instability of the DFE. □

Appendix B

Proof of Theorem 3.

Proof. Let $X = \mathcal{G}$, $x = (S_i, E_i, I_i, R_i, W_i)$ and $X_0 = \{x \in X | E_i + I_i + W_i > 0\}$. Hence, $\partial X_0 = X \setminus X_0 = \{x \in X | E_i = I_i = W_i = 0\}$. Let ψ_t be a semi-flow induced by the solutions of system (1) and $M_\partial = \{x \in \partial X_0 | \psi_t x \in \partial X_0, t \geq 0\}$. By Equation (2), we have $\psi_t X_0 \subset X_0$ and ψ_t is bounded in X_0. Therefore, there exist a global attractor for ψ_t. The disease-free equilibrium is the unique equilibrium on the manifold ∂X_0 and is globally asymptotically stable on ∂X_0. Moreover, $\bigcup_{x \in M_\partial} \omega(x) = \{\mathcal{E}^0\}$ and no subsets of M forms a cycle in ∂X_0. Finally, since the disease-free equilibrium is unstable on X_0 if $\mathcal{R}_0 > 1$, we deduce that system (1) is uniformly persistent by using a result from [6] (Theorem 1.3.1 and Remark 1.3.1). This completes the proof. □

Appendix C

Proof of Theorem 4.

We use the Centre Manifold Theory to examine the stability the endemic equilibrium point as presented in Theorem 4.1 Chavez and Song in 2004 [7], to ascertain the local asymptotic stability of the endemic equilibrium. We make the following changes on our original variables in order to apply the Centre Manifold Theory, $S_1 = x_1$, $E_1 = x_2$, $I_1 = x_3$, $R_1 = x_4$, $W_1 = x_5$, $S_2 = x_6$, $E_2 = x_7$, $I_2 = x_8$, $R_2 = x_9$, and $W_2 = x_{10}$. We now make use of the following vector notation $X = (x_1, x_2, x_3, x_4, x_5, x_6, x_7, x_8, x_9, x_{10})^T$. Thus, model system can now be presented in the form $\dfrac{dX}{dt} = F = (f_1, f_2, f_3, f_4, f_5, f_6, f_7, f_8, f_9, f_{10})^T$, such that

$$
\begin{aligned}
x_1' &= f_1 = b_1 - x_1 \left[\beta_1 p_{11} x_1 \left(\frac{p_{11}x_3 + p_{21}x_8}{p_{11}(x_1+x_2+x_3+x_4+x_5) + p_{21}(x_6+x_7+x_8+x_9+x_{10})} \right) + \right. \\
&\quad \left. \beta_2 p_{12} x_1 \left(\frac{p_{12}x_3 + p_{22}x_8}{p_{12}(x_1+x_2+x_3+x_4+x_5) + p_{22}(x_6+x_7+x_8+x_9+x_{10})} \right) \right] - B_1 p_{11} x_1 \frac{x_5}{K_1} - \mu_1 x_1 + \kappa_1 x_4, \\
x_2' &= f_2 = x_1 \left[\beta_1 p_{11} x_1 \left(\frac{p_{11}x_3 + p_{21}x_8}{p_{11}(x_1+x_2+x_3+x_4+x_5) + p_{21}(x_6+x_7+x_8+x_9+x_{10})} \right) + \right. \\
&\quad \left. \beta_2 p_{12} x_1 \left(\frac{p_{12}x_3 + p_{22}x_8}{p_{12}(x_1+x_2+x_3+x_4+x_5) + p_{22}(x_6+x_7+x_8+x_9+x_{10})} \right) \right] + B_1 p_{11} x_1 \frac{x_5}{K_1} - (\mu_1 + \omega_1) x_2, \\
x_3' &= f_3 = \omega_1 x_2 - (\mu_1 + \gamma_1 + v_1) x_3, \\
x_4' &= f_4 = \gamma_1 x_3 - (\mu_1 + \kappa_1) x_4, \\
x_5' &= f_5 = \eta_1 x_3 - r_1 x_5, \\
x_6' &= f_6 = b_2 - x_6 \left[\beta_1 p_{21} x_6 \left(\frac{p_{11}x_3 + p_{21}x_8}{p_{11}(x_1+x_2+x_3+x_4+x_5) + p_{21}(x_6+x_7+x_8+x_9+x_{10})} \right) + \right. \\
&\quad \left. \beta_2 p_{22} x_6 \left(\frac{p_{12}x_3 + p_{22}x_8}{p_{12}(x_1+x_2+x_3+x_4+x_5) + p_{22}(x_6+x_7+x_8+x_9+x_{10})} \right) \right] - B_2 p_{22} x_6 \frac{x_{10}}{K_2} - \mu_2 x_6 + \kappa_2 x_9, \\
x_7' &= f_7 = x_6 \left[\beta_1 p_{21} x_6 \left(\frac{p_{11}x_3 + p_{21}x_8}{p_{11}(x_1+x_2+x_3+x_4+x_5) + p_{21}(x_6+x_7+x_8+x_9+x_{10})} \right) + \right. \\
&\quad \left. \beta_2 p_{22} x_6 \left(\frac{p_{12}x_3 + p_{22}x_8}{p_{12}(x_1+x_2+x_3+x_4+x_5) + p_{22}(x_6+x_7+x_8+x_9+x_{10})} \right) \right] + B_2 p_{22} x_6 \frac{x_{10}}{K_2} - (\mu_2 + \omega_2) x_7, \\
x_8' &= f_8 = \omega_2 x_7 - (\mu_2 + \gamma_2 + v_2) x_8, \\
x_9' &= f_9 = \gamma_2 x_8 - (\mu_2 + \kappa_2) x_9, \\
x_{10}' &= f_{10} = \eta_2 x_8 - r_2 x_{10}.
\end{aligned} \tag{5}
$$

The method requires us to evaluate the Jacobian of the system (5) at the disease-free equilibrium as denoted by $\mathcal{E}^0$, with $S_1^0 = x_1^0$, $E_1^0 = x_2^0$, $I_1^0 = x_3^0$, $R_1^0 = x_4^0$, $W_1^0 = x_5^0$, $S_2^0 = x_6^0$, $E_2^0 = x_7^0$, $I_2^0 = x_8^0$, $R_2^0 = x_9^0$, and $W_2^0 = x_{10}^0$. Thus,

$$\mathcal{J}(\mathcal{E}^0) = \begin{pmatrix} \varphi_1 & 0 & -S_1^0\left[\frac{p_{11}^2 S_1^0\beta_1}{p_{11}S_1^0+p_{21}S_2^0}+\frac{p_{12}^2 S_1^0\beta_2}{p_{12}S_1^0+p_{22}S_2^0}\right] & 0 & -\frac{B_1 p_{11} S_1^0}{K_1} & 0 & 0 & -S_1^0\left[\frac{p_{11}p_{21}S_1^0\beta_1}{p_{11}S_1^0+p_{21}S_2^0}+\frac{p_{12}p_{22}S_1^0\beta_2}{p_{12}S_1^0+p_{22}S_2^0}\right] & 0 & 0 \\ 0 & -\mu_1-\omega_1 & S_1^0\left[\frac{p_{11}^2 S_1^0\beta_1}{p_{11}S_1^0+p_{21}S_2^0}+\frac{p_{12}^2 S_1^0\beta_2}{p_{12}S_1^0+p_{22}S_2^0}\right] & 0 & \frac{B_1 p_{11} S_1^0}{K_1} & 0 & 0 & S_1^0\left[\frac{p_{11}p_{21}S_1^0\beta_1}{p_{11}S_1^0+p_{21}S_2^0}+\frac{p_{12}p_{22}S_1^0\beta_2}{p_{12}S_1^0+p_{22}S_2^0}\right] & 0 & 0 \\ 0 & \omega_1 & -\phi_1 & 0 & 0 & 0 & 0 & 0 & 0 & 0 \\ 0 & 0 & \gamma_1 & -\varsigma_1 & 0 & 0 & 0 & 0 & 0 & 0 \\ 0 & 0 & \eta_1 & 0 & -r_1 & 0 & 0 & 0 & 0 & 0 \\ 0 & 0 & -S_2^0\left[\frac{p_{11}p_{21}S_2^0\beta_1}{p_{11}S_1^0+p_{21}S_2^0}+\frac{p_{12}p_{22}S_2^0\beta_2}{p_{12}S_1^0+p_{22}S_2^0}\right] & 0 & 0 & \varphi_2 & 0 & -S_2^0\left[\frac{p_{21}^2 S_2^0\beta_1}{p_{11}S_1^0+p_{21}S_2^0}+\frac{p_{22}^2 S_2^0\beta_2}{p_{12}S_1^0+p_{22}S_2^0}\right] & 0 & -\frac{B_2 p_{22} S_2^0}{K_2} \\ 0 & 0 & S_2^0\left[\frac{p_{11}p_{21}S_2^0\beta_1}{p_{11}S_1^0+p_{21}S_2^0}+\frac{p_{12}p_{22}S_2^0\beta_2}{p_{12}S_1^0+p_{22}S_2^0}\right] & 0 & 0 & 0 & -\mu_2-\omega_2 & S_2^0\left[\frac{p_{21}^2 S_2^0\beta_1}{p_{11}S_1^0+p_{21}S_2^0}+\frac{p_{22}^2 S_2^0\beta_2}{p_{12}S_1^0+p_{22}S_2^0}\right] & 0 & \frac{B_2 p_{22} S_2^0}{K_2} \\ 0 & 0 & 0 & 0 & 0 & 0 & \omega_2 & -\phi_2 & 0 & 0 \\ 0 & 0 & 0 & 0 & 0 & 0 & 0 & \gamma_2 & -\varsigma_2 & 0 \\ 0 & 0 & 0 & 0 & 0 & 0 & 0 & \eta_2 & 0 & -r_2 \end{pmatrix} \tag{6}$$

with $\varphi_i = \kappa_i - \mu_i > 0$ and $\varsigma_i = \kappa_i + \mu_i$, from which we can establish that

$$\mathcal{R}_0 = \frac{1}{2}\left(\frac{(M_1r_1 + M_2\eta_1)\omega_1}{a_1r_1\phi_1} + \frac{(M_5r_2 + M_6\eta_2)\omega_2}{a_2r_2\phi_2} + \sqrt{\begin{array}{c} \left(\frac{(M_1r_1 + M_2\eta_1)\omega_1}{a_1r_1\phi_1}\right)^2 + \left(\frac{(M_5r_2 + M_6\eta_2)\omega_2}{a_2r_2\phi_2}\right)^2 \\ -2\left(\frac{(M_1r_1 + M_2\eta_1)\omega_1}{a_1r_1\phi_1}\right)\left(\frac{(M_5r_2 + M_6\eta_2)\omega_2}{a_2r_2\phi_2}\right) \\ +4\left(\frac{\omega_1M_3}{a_1\phi_1}\right)\left(\frac{\omega_2M_4}{a_2\phi_2}\right) \end{array}} \right), \quad (7)$$

where

$$M_1 = \frac{p_{11}^2\beta_1N_1}{p_{11}N_1 + p_{21}N_2} + \frac{p_{12}^2\beta_2N_1}{p_{12}N_1 + p_{22}N_2}, \quad M_2 = B_1p_{11}\frac{N_1}{K_1}, \quad M_3 = \frac{p_{11}p_{21}\beta_1N_1}{p_{11}N_1 + p_{12}N_2} + \frac{p_{12}p_{22}\beta_2N_1}{p_{12}N_1 + p_{22}N_2},$$

$$M_4 = \frac{p_{11}p_{21}\beta_1N_2}{p_{11}N_1 + p_{12}N_2} + \frac{p_{12}p_{22}\beta_2N_2}{p_{12}N_1 + p_{22}N_2}, \quad M_5 = \frac{p_{21}^2\beta_1N_2}{p_{11}N_1 + p_{21}N_2} + \frac{p_{22}^2\beta_2N_2}{p_{12}N_1 + p_{22}N_2}, \quad M_6 = B_2p_{22}\frac{N_2}{K_2},$$

$$a_1 = \mu_1+\omega_1, \quad a_2 = \mu_2+\omega_2, \quad \phi_1 = \mu_1+v_1+\gamma_1, \quad \phi_2 = \mu_2+v_2+\gamma_2. \quad (8)$$

Considering $\beta_2 = \rho_0\beta_1,\ B_1 = \rho_1\beta_1\ B_2 = \rho_2\beta_1$ regardless of whether $\rho_i \in (0,1)$ or $\rho_i \geq 1$ for $i = 0, 1,$ and 2. Considering the case for $\mathcal{R}_0 = 1$ and taking β_1 as our bifurcation parameter and solving for β_1, we obtain

$$\beta^* = \beta_1 = \frac{2}{\left(\frac{(T_1r_1 + T_2\eta_1)\omega_1}{a_1r_1\phi_1} + \frac{(T_5r_2 + T_6\eta_2)\omega_2}{a_2r_2\phi_2} + \sqrt{\begin{array}{c} \left(\frac{(T_1r_1 + T_2\eta_1)\omega_1}{a_1r_1\phi_1}\right)^2 + \left(\frac{(T_5r_2 + T_6\eta_2)\omega_2}{a_2r_2\phi_2}\right)^2 \\ -2\left(\frac{(T_1r_1 + T_2\eta_1)\omega_1}{a_1r_1\phi_1}\right)\left(\frac{(T_5r_2 + T_6\eta_2)\omega_2}{a_2r_2\phi_2}\right) \\ +4\left(\frac{\omega_1T_3}{a_1\phi_1}\right)\left(\frac{\omega_2T_4}{a_2\phi_2}\right) \end{array}} \right)},$$

where

$$T_1 = \frac{p_{11}^2\beta_1N_1}{p_{11}N_1 + p_{21}N_2} + \frac{p_{12}^2\rho_0\beta_1N_1}{p_{12}N_1 + p_{22}N_2}, \quad T_2 = \rho_1\beta_1p_{11}\frac{N_1}{K_1}, \quad T_3 = \frac{p_{11}p_{21}\beta_1N_1}{p_{11}N_1 + p_{12}N_2} + \frac{p_{12}p_{22}\rho_1\beta_1N_1}{p_{12}N_1 + p_{22}N_2},$$

$$T_4 = \frac{p_{11}p_{21}\beta_1N_2}{p_{11}N_1 + p_{12}N_2} + \frac{p_{12}p_{22}\rho_1\beta_1N_2}{p_{12}N_1 + p_{22}N_2}, \quad T_5 = \frac{p_{21}^2\beta_1N_2}{p_{11}N_1 + p_{21}N_2} + \frac{p_{22}^2\rho_0\beta_1N_2}{p_{12}N_1 + p_{22}N_2}, \quad T_6 = \rho_2\beta_1p_{22}\frac{N_2}{K_2},$$

$$a_1 = \mu_1 + \omega_1, \quad a_2 = \mu_2 + \omega_2, \quad \phi_1 = \mu_1 + v_1 + \gamma_1, \quad \phi_2 = \mu_2 + v_2 + \gamma_2. \quad (9)$$

It is worth noting that the linearized system of our transformed equation with the bifurcation point β^* comprises of a simple eigenvalue. Therefore, we can apply the center manifold theory to analyze the dynamics of the system near $\beta^* = \beta_1$. It can be demonstrated that the Jacobian of our model system has a right

eigenvalue related with the following zero eigenvalue $u = (u_1, u_2, u_3, u_4, u_5, u_6, u_7, u_8, u_9, u_{10})^T$, where

$$\begin{cases} u_1 = -\dfrac{u_3 S_1^0}{\varphi_1}\left[\dfrac{p_{11}^2 S_1^0 \beta_1}{p_{11}S_1^0 + p_{21}S_2^0} + \dfrac{p_{12}^2 S_1^0 \beta_2}{p_{12}S_1^0 + p_{22}S_2^0}\right] - \dfrac{u_8 S_1^0}{\varphi_1}\left[\dfrac{p_{11}p_{21}S_1^0\beta_1}{p_{11}S_1^0 + p_{21}S_2^0} + \dfrac{p_{12}p_{22}S_1^0\beta_2}{p_{12}S_1^0 + p_{22}S_2^0}\right] \\ \quad - \dfrac{\rho_1\beta_1 p_{11} S_1^0 \eta_1 u_3}{K_1 r_1 \varphi_1} < 0, \\ u_2 = \dfrac{\phi_1 u_3}{\omega_1} > 0, \quad u_3 > 0, \quad u_4 = \dfrac{\phi_1 u_3}{\mu_1} > 0, \quad u_5 = \dfrac{\eta_1 u_3}{r_1} > 0, \\ u_6 = -\dfrac{u_8 S_2^0}{\varphi_2}\left[\dfrac{p_{21}^2 S_2^0 \beta_1}{p_{11}S_1^0 + p_{21}S_2^0} + \dfrac{p_{22}^2 S_2^0 \beta_2}{p_{12}S_1^0 + p_{22}S_2^0}\right] - \dfrac{u_3 S_2^0}{\varphi_2}\left[\dfrac{p_{11}p_{21}S_2^0\beta_1}{p_{11}S_1^0 + p_{21}S_2^0} + \dfrac{p_{12}p_{22}S_2^0\beta_2}{p_{12}S_1^0 + p_{22}S_2^0}\right] \\ \quad - \dfrac{u_8\eta_2\rho_2\beta_1 p_{22} S_2^0}{K_2 r_2 \varphi_2} < 0, \\ u_7 = \dfrac{\phi_2 u_8}{\omega_2} > 0, \quad u_8 > 0, \quad u_9 = \dfrac{\phi_2 u_8}{\mu_2} > 0, \quad u_{10} = \dfrac{\eta_2 u_8}{r_2} > 0. \end{cases} \tag{10}$$

The left eigenvector of $\mathcal{J}(\mathcal{E}^0)$ associated with the zero eigenvalue at $\beta^* = \beta_1$ is given by

$$v = (v_1, v_2, v_3, v_4, v_5, v_6, v_7, v_8, v_9, v_{10})^T,$$

where

$$\begin{cases} v_1 = 0, \quad v_2 = \dfrac{\omega_1 v_3}{\mu_1 + \omega_1} > 0, \quad v_3 > 0, \quad v_4 = 0, \quad v_5 = \dfrac{\rho_1\beta_1 p_{11} S_1^0 v_3 \omega_1}{r_1 K_1(\mu_1 + \omega_1)} > 0, \\ v_6 = 0, \quad v_7 = \dfrac{\omega_2 v_8}{\mu_2 + \omega_2} > 0, \quad v_8 > 0, \quad v_9 = 0, \quad v_{10} = \dfrac{\rho_2\beta_1 p_{22} S_2^0 v_8 \omega_2}{r_2 K_2(\mu_2 + \omega_2)} > 0. \end{cases} \tag{11}$$

Computation of the bifurcation parameters a and b

For model system (5), the respective non-zero partial derivatives of F at the infection-free equilibrium are given by:

$$\begin{aligned} \frac{\partial^2 f_1}{\partial x_1 \partial x_3} &= \frac{\partial^2 f_1}{\partial x_3 \partial x_1} = -S_1^0\left[-\frac{p_{11}^3 S_1^0 \beta_1}{(p_{11}S_1^0 + p_{21}S_2^0)^2} + \frac{2p_{11}^2\beta_1}{p_{11}S_1^0 + p_{21}S_2^0} - \frac{p_{12}^3 S_1^0 \beta_2}{(p_{12}S_1^0 + p_{22}S_2^0)^2} + \frac{2p_{12}^2\beta_2}{p_{12}S_1^0 + p_{22}S_2^0}\right], \\ \frac{\partial^2 f_1}{\partial x_1 \partial x_5} &= \frac{\partial^2 f_1}{\partial x_5 \partial x_1} = -\frac{B_1 p_{11}}{K_1}, \\ \frac{\partial^2 f_1}{\partial x_1 \partial x_8} &= \frac{\partial^2 f_1}{\partial x_8 \partial x_1} = -S_1^0\left[-\frac{p_{11}^2 p_{21} S_1^0 \beta_1}{(p_{11}S_1^0 + p_{21}S_2^0)^2} + \frac{2p_{11}p_{21}\beta_1}{p_{11}S_1^0 + p_{21}S_2^0} - \frac{p_{12}^2 p_{22} S_1^0 \beta_2}{(p_{12}S_1^0 + p_{22}S_2^0)^2} + \frac{2p_{12}p_{22}\beta_2}{p_{12}S_1^0 + p_{22}S_2^0}\right], \\ \frac{\partial^2 f_1}{\partial x_2 \partial x_3} &= \frac{\partial^2 f_1}{\partial x_3 \partial x_2} = \frac{1}{2}\frac{\partial^2 f_1}{\partial x_3 \partial x_3} = \frac{\partial^2 f_1}{\partial x_3 \partial x_4} = \frac{\partial^2 f_1}{\partial x_3 \partial x_5} = \frac{\partial^2 f_1}{\partial x_4 \partial x_3} = \frac{\partial^2 f_1}{\partial x_5 \partial x_3} \\ &= S_1^0\left[\frac{p_{11}^3 S_1^0 \beta_1}{(p_{11}S_1^0 + p_{21}S_2^0)^2} + \frac{p_{12}^3 S_1^0 \beta_2}{(p_{12}S_1^0 + p_{22}S_2^0)^2}\right], \end{aligned}$$

$$
\begin{aligned}
\frac{\partial^2 f_1}{\partial x_2 \partial x_8} &= \frac{\partial^2 f_1}{\partial x_3 \partial x_6} = \frac{\partial^2 f_1}{\partial x_3 \partial x_7} = \frac{1}{2}\frac{\partial^2 f_1}{\partial x_3 \partial x_8} = \frac{\partial^2 f_1}{\partial x_3 \partial x_9} = \frac{\partial^2 f_1}{\partial x_3 \partial x_{10}} = \frac{\partial^2 f_1}{\partial x_4 \partial x_8} = \frac{\partial^2 f_1}{\partial x_5 \partial x_8} = \frac{\partial^2 f_1}{\partial x_6 \partial x_3} = \frac{\partial^2 f_1}{\partial x_7 \partial x_3} \\
&= \frac{\partial^2 f_1}{\partial x_8 \partial x_2} = \frac{1}{2}\frac{\partial^2 f_1}{\partial x_8 \partial x_3} = \frac{\partial^2 f_1}{\partial x_8 \partial x_4} = \frac{\partial^2 f_1}{\partial x_8 \partial x_5} = \frac{\partial^2 f_1}{\partial x_9 \partial x_3} = \frac{\partial^2 f_1}{\partial x_{10} \partial x_3} \\
&= S_1^0 \left[\frac{p_{11}^2 p_{21} S_1^0 \beta_1}{(p_{11} S_1^0 + p_{21} S_2^0)^2} + \frac{p_{12}^2 p_{22} S_1^0 \beta_2}{(p_{12} S_1^0 + p_{22} S_2^0)^2} \right], \\
\frac{\partial^2 f_1}{\partial x_6 \partial x_8} &= \frac{\partial^2 f_1}{\partial x_7 \partial x_8} = \frac{\partial^2 f_1}{\partial x_8 \partial x_6} = \frac{\partial^2 f_1}{\partial x_8 \partial x_7} = \frac{1}{2}\frac{\partial^2 f_1}{\partial x_8 \partial x_8} = \frac{\partial^2 f_1}{\partial x_8 \partial x_9} = \frac{\partial^2 f_1}{\partial x_8 \partial x_{10}} = \frac{\partial^2 f_1}{\partial x_9 \partial x_8} = \frac{\partial^2 f_1}{\partial x_{10} \partial x_8} \\
&= S_1^0 \left[\frac{p_{11} p_{21}^2 S_1^0 \beta_1}{(p_{11} S_1^0 + p_{21} S_2^0)^2} + \frac{p_{12} p_{22}^2 S_1^0 \beta_2}{(p_{12} S_1^0 + p_{22} S_2^0)^2} \right], \\
\frac{\partial^2 f_2}{\partial x_1 \partial x_3} &= \frac{\partial^2 f_2}{\partial x_3 \partial x_1} = S_1^0 \left[-\frac{p_{11}^3 S_1^0 \beta_1}{(p_{11} S_1^0 + p_{21} S_2^0)^2} + \frac{2 p_{11}^2 \beta_1}{p_{11} S_1^0 + p_{21} S_2^0} - \frac{p_{12}^3 S_1^0 \beta_2}{(p_{12} S_1^0 + p_{22} S_2^0)^2} + \frac{2 p_{12}^2 \beta_2}{p_{12} S_1^0 + p_{22} S_2^0} \right], \\
\frac{\partial^2 f_2}{\partial x_1 \partial x_5} &= \frac{\partial^2 f_2}{\partial x_5 \partial x_1} = \frac{B_1 p_{11}}{K_1},
\end{aligned}
$$

$$
\begin{aligned}
\frac{\partial^2 f_2}{\partial x_1 \partial x_8} &= \frac{\partial^2 f_2}{\partial x_8 \partial x_1} = S_1^0 \left[-\frac{p_{11}^2 p_{21} S_1^0 \beta_1}{(p_{11} S_1^0 + p_{21} S_2^0)^2} + \frac{2 p_{11} p_{21} \beta_1}{p_{11} S_1^0 + p_{21} S_2^0} - \frac{p_{12}^2 p_{22} S_1^0 \beta_2}{(p_{12} S_1^0 + p_{22} S_2^0)^2} + \frac{2 p_{12} p_{22} \beta_2}{p_{12} S_1^0 + p_{22} S_2^0} \right], \\
\frac{\partial^2 f_2}{\partial x_2 \partial x_3} &= \frac{\partial^2 f_2}{\partial x_3 \partial x_2} = \frac{1}{2}\frac{\partial^2 f_2}{\partial x_3 \partial x_3} = \frac{\partial^2 f_2}{\partial x_3 \partial x_4} = \frac{\partial^2 f_2}{\partial x_3 \partial x_5} = \frac{\partial^2 f_2}{\partial x_4 \partial x_3} = \frac{\partial^2 f_2}{\partial x_5 \partial x_3} \\
&= -S_1^0 \left[\frac{p_{11}^3 S_1^0 \beta_1}{(p_{11} S_1^0 + p_{21} S_2^0)^2} + \frac{p_{12}^3 S_1^0 \beta_2}{(p_{12} S_1^0 + p_{22} S_2^0)^2} \right], \\
\frac{\partial^2 f_2}{\partial x_2 \partial x_8} &= \frac{\partial^2 f_2}{\partial x_3 \partial x_6} = \frac{\partial^2 f_2}{\partial x_3 \partial x_7} = \frac{1}{2}\frac{\partial^2 f_2}{\partial x_3 \partial x_8} = \frac{\partial^2 f_2}{\partial x_3 \partial x_9} = \frac{\partial^2 f_2}{\partial x_3 \partial x_{10}} = \frac{\partial^2 f_2}{\partial x_4 \partial x_8} = \frac{\partial^2 f_2}{\partial x_5 \partial x_8} = \frac{\partial^2 f_2}{\partial x_6 \partial x_3} = \frac{\partial^2 f_2}{\partial x_7 \partial x_3} \\
&= \frac{\partial^2 f_2}{\partial x_8 \partial x_2} = \frac{1}{2}\frac{\partial^2 f_2}{\partial x_8 \partial x_3} = \frac{\partial^2 f_2}{\partial x_8 \partial x_4} = \frac{\partial^2 f_2}{\partial x_8 \partial x_5} = \frac{\partial^2 f_2}{\partial x_9 \partial x_3} = \frac{\partial^2 f_2}{\partial x_{10} \partial x_3} \\
&= -S_1^0 \left[\frac{p_{11}^2 p_{21} S_1^0 \beta_1}{(p_{11} S_1^0 + p_{21} S_2^0)^2} + \frac{p_{12}^2 p_{22} S_1^0 \beta_2}{(p_{12} S_1^0 + p_{22} S_2^0)^2} \right],
\end{aligned}
$$

$$\begin{aligned}
\frac{\partial^2 f_2}{\partial x_6 \partial x_8} &= \frac{\partial^2 f_2}{\partial x_7 \partial x_8} = \frac{\partial^2 f_2}{\partial x_8 \partial x_6} = \frac{\partial^2 f_2}{\partial x_8 \partial x_7} = \frac{1}{2}\frac{\partial^2 f_2}{\partial x_8 \partial x_8} = \frac{\partial^2 f_2}{\partial x_8 \partial x_9} = \frac{\partial^2 f_2}{\partial x_8 \partial x_{10}} = \frac{\partial^2 f_2}{\partial x_9 \partial x_8} = \frac{\partial^2 f_2}{\partial x_{10} \partial x_8} \\
&= -S_1^0\left[\frac{p_{11}p_{21}^2 S_1^0 \beta_1}{(p_{11}S_1^0 + p_{21}S_2^0)^2} + \frac{p_{12}p_{22}^2 S_1^0 \beta_2}{(p_{12}S_1^0 + p_{22}S_2^0)^2}\right], \\
\frac{\partial^2 f_6}{\partial x_1 \partial x_3} &= \frac{\partial^2 f_6}{\partial x_2 \partial x_3} = \frac{\partial^2 f_6}{\partial x_3 \partial x_1} = \frac{\partial^2 f_6}{\partial x_3 \partial x_2} = \frac{1}{2}\frac{\partial^2 f_6}{\partial x_3 \partial x_3} = \frac{\partial^2 f_6}{\partial x_3 \partial x_4} = \frac{\partial^2 f_6}{\partial x_3 \partial x_5} = \frac{\partial^2 f_6}{\partial x_4 \partial x_3} = \frac{\partial^2 f_6}{\partial x_5 \partial x_3} \\
&= S_2^0\left[\frac{p_{11}^2 p_{21} S_2^0 \beta_1}{(p_{11}S_1^0 + p_{21}S_2^0)^2} + \frac{p_{12}^2 p_{22} S_2^0 \beta_2}{(p_{12}S_1^0 + p_{22}S_2^0)^2}\right], \\
\frac{\partial^2 f_6}{\partial x_1 \partial x_8} &= \frac{\partial^2 f_6}{\partial x_2 \partial x_8} = \frac{\partial^2 f_6}{\partial x_3 \partial x_7} = \frac{1}{2}\frac{\partial^2 f_6}{\partial x_3 \partial x_8} = \frac{\partial^2 f_6}{\partial x_3 \partial x_9} = \frac{\partial^2 f_6}{\partial x_3 \partial x_{10}} = \frac{\partial^2 f_6}{\partial x_4 \partial x_8} = \frac{\partial^2 f_6}{\partial x_5 \partial x_8} = \frac{\partial^2 f_6}{\partial x_7 \partial x_3} = \frac{\partial^2 f_6}{\partial x_8 \partial x_1} \\
&= \frac{\partial^2 f_6}{\partial x_8 \partial x_2} = \frac{1}{2}\frac{\partial^2 f_6}{\partial x_8 \partial x_3} = \frac{\partial^2 f_6}{\partial x_8 \partial x_4} = \frac{\partial^2 f_6}{\partial x_8 \partial x_5} = \frac{\partial^2 f_6}{\partial x_9 \partial x_3} = \frac{\partial^2 f_6}{\partial x_{10} \partial x_3} \\
&= S_2^0\left[\frac{p_{11}p_{21}^2 S_2^0 \beta_1}{(p_{11}S_1^0 + p_{21}S_2^0)^2} + \frac{p_{12}p_{22}^2 S_2^0 \beta_2}{(p_{12}S_1^0 + p_{22}S_2^0)^2}\right],
\end{aligned}$$

$$\begin{aligned}
\frac{\partial^2 f_6}{\partial x_3 \partial x_6} &= \frac{\partial^2 f_6}{\partial x_6 \partial x_3} = -S_2^0\left[-\frac{p_{11}p_{21}^2 S_2^0 \beta_1}{(p_{11}S_1^0 + p_{21}S_2^0)^2} + \frac{2p_{11}p_{21}\beta_1}{p_{11}S_1^0 + p_{21}S_2^0} - \frac{p_{12}p_{22}^2 S_2^0 \beta_2}{(p_{12}S_1^0 + p_{22}S_2^0)^2} + \frac{2p_{12}p_{22}\beta_2}{p_{12}S_1^0 + p_{22}S_2^0}\right], \\
\frac{\partial^2 f_6}{\partial x_6 \partial x_8} &= \frac{\partial^2 f_6}{\partial x_8 \partial x_6} = -S_2^0\left[-\frac{p_{21}^3 S_2^0 \beta_1}{(p_{11}S_1^0 + p_{21}S_2^0)^2} + \frac{2p_{21}^2\beta_1}{p_{11}S_1^0 + p_{21}S_2^0} - \frac{p_{22}^3 S_2^0 \beta_2}{(p_{12}S_1^0 + p_{22}S_2^0)^2} + \frac{2p_{22}^2\beta_2}{p_{12}S_1^0 + p_{22}S_2^0}\right], \\
\frac{\partial^2 f_6}{\partial x_6 \partial x_{10}} &= \frac{\partial^2 f_6}{\partial x_{10} \partial x_6} = -\frac{B_2 p_{22}}{K_2} \\
\frac{\partial^2 f_6}{\partial x_7 \partial x_8} &= \frac{\partial^2 f_6}{\partial x_8 \partial x_7} = \frac{1}{2}\frac{\partial^2 f_6}{\partial x_8 \partial x_8} = \frac{\partial^2 f_6}{\partial x_8 \partial x_9} = \frac{\partial^2 f_2}{\partial x_8 \partial x_{10}} = \frac{\partial^2 f_6}{\partial x_9 \partial x_8} = \frac{\partial^2 f_6}{\partial x_{10} \partial x_8} \\
&= S_2^0\left[\frac{p_{21}^3 S_2^0 \beta_1}{(p_{11}S_1^0 + p_{21}S_2^0)^2} + \frac{p_{22}^3 S_2^0 \beta_2}{(p_{12}S_1^0 + p_{22}S_2^0)^2}\right],
\end{aligned}$$

$$\begin{aligned}
\frac{\partial^2 f_7}{\partial x_1 \partial x_3} &= \frac{\partial^2 f_7}{\partial x_2 \partial x_3} = \frac{\partial^2 f_7}{\partial x_3 \partial x_1} = \frac{\partial^2 f_7}{\partial x_3 \partial x_2} = \frac{1}{2}\frac{\partial^2 f_7}{\partial x_3 \partial x_3} = \frac{\partial^2 f_7}{\partial x_3 \partial x_4} = \frac{\partial^2 f_7}{\partial x_3 \partial x_5} = \frac{\partial^2 f_7}{\partial x_4 \partial x_3} = \frac{\partial^2 f_7}{\partial x_5 \partial x_3} \\
&= -S_2^0\left[\frac{p_{11}^2 p_{21} S_2^0 \beta_1}{(p_{11}S_1^0 + p_{21}S_2^0)^2} + \frac{p_{12}^2 p_{22} S_2^0 \beta_2}{(p_{12}S_1^0 + p_{22}S_2^0)^2}\right], \\
\frac{\partial^2 f_7}{\partial x_1 \partial x_8} &= \frac{\partial^2 f_7}{\partial x_2 \partial x_8} = \frac{\partial^2 f_7}{\partial x_3 \partial x_7} = \frac{1}{2}\frac{\partial^2 f_7}{\partial x_3 \partial x_8} = \frac{\partial^2 f_7}{\partial x_3 \partial x_9} = \frac{\partial^2 f_7}{\partial x_3 \partial x_{10}} = \frac{\partial^2 f_7}{\partial x_4 \partial x_8} = \frac{\partial^2 f_7}{\partial x_5 \partial x_8} = \frac{\partial^2 f_7}{\partial x_7 \partial x_3} = \frac{\partial^2 f_7}{\partial x_8 \partial x_1} \\
&= \frac{\partial^2 f_7}{\partial x_8 \partial x_2} = \frac{1}{2}\frac{\partial^2 f_7}{\partial x_8 \partial x_3} = \frac{\partial^2 f_7}{\partial x_8 \partial x_4} = \frac{\partial^2 f_7}{\partial x_8 \partial x_5} = \frac{\partial^2 f_7}{\partial x_9 \partial x_3} = \frac{\partial^2 f_7}{\partial x_{10} \partial x_3} \\
&= -S_2^0\left[\frac{p_{11}p_{21}^2 S_2^0 \beta_1}{(p_{11}S_1^0 + p_{21}S_2^0)^2} + \frac{p_{12}p_{22}^2 S_2^0 \beta_2}{(p_{12}S_1^0 + p_{22}S_2^0)^2}\right],
\end{aligned}$$

$$\begin{aligned}
\frac{\partial^2 f_7}{\partial x_3 \partial x_6} &= \frac{\partial^2 f_7}{\partial x_6 \partial x_3} = S_2^0\left[-\frac{p_{11}p_{21}^2 S_2^0 \beta_1}{(p_{11}S_1^0 + p_{21}S_2^0)^2} + \frac{2p_{11}p_{21}\beta_1}{p_{11}S_1^0 + p_{21}S_2^0} - \frac{p_{12}p_{22}^2 S_2^0 \beta_2}{(p_{12}S_1^0 + p_{22}S_2^0)^2} + \frac{2p_{12}p_{22}\beta_2}{p_{12}S_1^0 + p_{22}S_2^0}\right] \\
\frac{\partial^2 f_7}{\partial x_6 \partial x_8} &= \frac{\partial^2 f_7}{\partial x_8 \partial x_6} = S_2^0\left[-\frac{p_{21}^3 S_2^0 \beta_1}{(p_{11}S_1^0 + p_{21}S_2^0)^2} + \frac{2p_{21}^2\beta_1}{p_{11}S_1^0 + p_{21}S_2^0} - \frac{p_{22}^3 S_2^0 \beta_2}{(p_{12}S_1^0 + p_{22}S_2^0)^2} + \frac{2p_{22}^2\beta_2}{p_{12}S_1^0 + p_{22}S_2^0}\right],
\end{aligned}$$

$$\begin{aligned}
\frac{\partial^2 f_7}{\partial x_6 \partial x_{10}} &= \frac{\partial^2 f_7}{\partial x_{10} \partial x_6} = \frac{B_2 p_{22}}{K_2}, \\
\frac{\partial^2 f_7}{\partial x_7 \partial x_8} &= \frac{\partial^2 f_7}{\partial x_8 \partial x_7} = \frac{1}{2} \frac{\partial^2 f_7}{\partial x_8 \partial x_8} = \frac{\partial^2 f_7}{\partial x_8 \partial x_9} = \frac{\partial^2 f_7}{\partial x_8 \partial x_{10}} = \frac{\partial^2 f_7}{\partial x_9 \partial x_8} = \frac{\partial^2 f_7}{\partial x_{10} \partial x_8} \\
&= -S_2^0 \left[\frac{p_{21}^3 S_2^0 \beta_1}{(p_{11} S_1^0 + p_{21} S_2^0)^2} + \frac{p_{22}^3 S_2^0 \beta_2}{(p_{12} S_1^0 + p_{22} S_2^0)^2} \right]. \qquad (12)
\end{aligned}$$

From (12) we have that

$$\begin{aligned}
a &= -2v_2(u_6 + u_7 + u_8 + u_9 + u_{10})(u_3 \mathcal{W}_1 + u_8 \mathcal{W}_2) - 2v_2 u_8 \mathcal{W}_1 (u_2 + u_4 + u_5) \\
&\quad -2v_2 u_3 \mathcal{W}_6 (u_2 + u_3 + u_4 + u_5) + 2v_2 u_1 (u_3 \mathcal{W}_3 + u_8 \mathcal{W}_4 + u_{10} \mathcal{W}_5) \\
&\quad -2v_7(u_1 + u_2 + u_3 + u_4 + u_5)(u_3 \mathcal{Z}_1 + u_8 \mathcal{Z}_2) - 2v_7 u_3 \mathcal{Z}_2 (u_7 + u_9 + u_{10}) \\
&\quad -2v_7 u_8 \mathcal{Z}_6 (u_7 + u_8 + u_9 + u_{10}) + 2v_7 u_6 (u_3 \mathcal{Z}_3 + u_8 \mathcal{Z}_4 + u_{10} \mathcal{Z}_5) \\
&\quad < 0,
\end{aligned}$$

where

$$\mathcal{W}_1 = S_1^0 \left[\frac{p_{11}^2 p_{21} S_1^0 \beta_1}{(p_{11} S_1^0 + p_{21} S_2^0)^2} + \frac{p_{12}^2 p_{22} S_1^0 \beta_2}{(p_{12} S_1^0 + p_{22} S_2^0)^2} \right], \quad \mathcal{W}_2 = S_1^0 \left[\frac{p_{11} p_{21}^2 S_1^0 \beta_1}{(p_{11} S_1^0 + p_{21} S_2^0)^2} + \frac{p_{12} p_{22}^2 S_1^0 \beta_2}{(p_{12} S_1^0 + p_{22} S_2^0)^2} \right],$$

$$\mathcal{W}_3 = S_1^0 \left[\frac{p_{11}^2 \beta_1}{(p_{11} S_1^0 + p_{21} S_2^0)} \left(2 - \frac{p_{11} S_1^0}{p_{11} S_1^0 + p_{21} S_2^0} \right) + \frac{p_{12}^2 \beta_2}{(p_{12} S_1^0 + p_{22} S_2^0)} \left(2 - \frac{p_{12} S_1^0}{p_{12} S_1^0 + p_{22} S_2^0} \right) \right],$$

$$\mathcal{W}_4 = S_1^0 \left[\frac{p_{11} p_{12} \beta_1}{(p_{11} S_1^0 + p_{21} S_2^0)} \left(2 - \frac{p_{11} S_1^0}{p_{11} S_1^0 + p_{21} S_2^0} \right) + \frac{p_{12} p_{22} \beta_2}{(p_{12} S_1^0 + p_{22} S_2^0)} \left(2 - \frac{p_{12} S_1^0}{p_{12} S_1^0 + p_{22} S_2^0} \right) \right], \quad \mathcal{W}_5 = \frac{p_{11} B_1}{K_1},$$

$$\mathcal{Z}_1 = S_2^0 \left[\frac{p_{11}^2 p_{21} S_2^0 \beta_1}{(p_{11} S_1^0 + p_{21} S_2^0)^2} + \frac{p_{12}^2 p_{22} S_2^0 \beta_2}{(p_{12} S_1^0 + p_{22} S_2^0)^2} \right], \quad \mathcal{Z}_2 = S_2^0 \left[\frac{p_{11} p_{21}^2 S_2^0 \beta_1}{(p_{11} S_1^0 + p_{21} S_2^0)^2} + \frac{p_{12} p_{22}^2 S_2^0 \beta_2}{(p_{12} S_1^0 + p_{22} S_2^0)^2} \right],$$

$$\mathcal{Z}_4 = S_2^0 \left[\frac{p_{21}^2 \beta_1}{(p_{11} S_1^0 + p_{21} S_2^0)} \left(2 - \frac{p_{21} S_2^0}{p_{11} S_1^0 + p_{21} S_2^0} \right) + \frac{p_{22}^2 \beta_2}{(p_{22} S_2^0 + p_{22} S_2^0)} \left(2 - \frac{p_{12} S_1^0}{p_{12} S_1^0 + p_{22} S_2^0} \right) \right],$$

$$\mathcal{Z}_3 = S_1^0 \left[\frac{p_{11} p_{21} \beta_1}{(p_{11} S_1^0 + p_{21} S_2^0)} \left(2 - \frac{p_{11} S_2^0}{p_{11} S_1^0 + p_{21} S_2^0} \right) + \frac{p_{12} p_{22} \beta_2}{(p_{12} S_1^0 + p_{22} S_2^0)} \left(2 - \frac{p_{22} S_2^0}{p_{12} S_1^0 + p_{22} S_2^0} \right) \right], \quad \mathcal{Z}_5 = \frac{p_{22} B_2}{K_2}.$$

$$\begin{aligned}
\frac{\partial^2 f_2}{\partial x_3 \partial x_{\beta_1}} &= S_1^0 \left(\frac{p_{11}^2 S_1^0}{p_{11} S_1^0 + p_{21} S_2^0} + \frac{\rho_0 p_{12}^2 S_1^0}{p_{12} S_1^0 + p_{22} S_2^0} \right), \quad \frac{\partial^2 f_2}{\partial x_8 \partial x_{\beta_1}} = S_1^0 \left(\frac{p_{11} p_{21}^2 S_1^0}{p_{11} S_1^0 + p_{12} S_2^0} + \frac{\rho_0 p_{12} p_{22} S_1^0}{p_{12} S_1 + p_{22} S_2} \right), \\
\frac{\partial^2 f_7}{\partial x_3 \partial x_{\beta_1}} &= S_2^0 \left(\frac{p_{11} p_{21} S_2^0}{p_{11} S_1 + p_{12} S_2} + \frac{\rho_0 p_{12} p_{22} S_2^0}{p_{12} S_1 + p_{22} S_2} \right), \quad \frac{\partial^7 f_2}{\partial x_8 \partial x_{\beta_1}} = S_2^0 \left(\frac{p_{21}^2 S_2^0}{p_{11} S_1^0 + p_{21} S_2^0} + \frac{\rho_0 p_{12}^2 S_2^0}{p_{12} S_1^0 + p_{22} S_2^0} \right), \\
\frac{\partial^2 f_2}{\partial x_5 \partial x_{\beta_1}} &= \frac{\rho_1 p_{11} S_1^0}{K_1}, \quad \frac{\partial^2 f_7}{\partial x_{10} \partial x_{\beta_1}} = \frac{\rho_2 p_{22} S_2^0}{K_2}. \qquad (13)
\end{aligned}$$

From the expressions in (13), it follows that

$$b = v_2(u_3\mathcal{S}_1 + u_5\mathcal{S}_2 + u_7\mathcal{S}_3) + v_7(u_3\mathcal{T}_1 + u_5\mathcal{T}_2 + u_{10}\mathcal{T}_3), \tag{14}$$

where

$$\begin{aligned}
\mathcal{S}_1 &= S_1^0\left(\frac{p_{11}^2 S_1^0}{p_{11}S_1^0 + p_{21}S_2^0} + \frac{\rho_0 p_{12}^2 S_1^0}{p_{12}S_1^0 + p_{22}S_2^0}\right), \quad \mathcal{T}_1 = S_1^0\left(\frac{p_{11}p_{21}^2 S_1^0}{p_{11}S_1^0 + p_{12}S_2^0} + \frac{\rho_0 p_{12}p_{22}S_1^0}{p_{12}S_1 + p_{22}S_2}\right), \\
\mathcal{S}_2 &= S_2^0\left(\frac{p_{11}p_{21}S_2^0}{p_{11}S_1 + p_{12}S_2} + \frac{\rho_0 p_{12}p_{22}S_2^0}{p_{12}S_1 + p_{22}S_2}\right), \quad \mathcal{T}_2 = S_2^0\left(\frac{p_{21}^2 S_2^0}{p_{11}S_1^0 + p_{21}S_2^0} + \frac{\rho_0 p_{12}^2 S_2^0}{p_{12}S_1^0 + p_{22}S_2^0}\right), \\
\mathcal{S}_3 &= \frac{\rho_1 p_{11}S_1^0}{K_1}, \quad \mathcal{T}_3 = \frac{\rho_2 p_{22}S_2^0}{K_2}.
\end{aligned}$$

Hence, we have established that $a < 0$ and $b > 0$. Thus Theorem 4 has been established.

References

[1] Horn, R. A. & Johnson C. R. Matrix Analysis. (Cambridge University Press, 1985).

[2] Shuai, Z., Heesterbeek, J.A.P. & van den Driessche, P. Extending the type reproduction number to infectious disease control targeting contact between types. *J Math Biol.* **67**(5), 1067-1082 (2013) .

[3] LaSalle, J.P. The Stability of Dynamical Systems, in: CBMS-NSF Regional Conference Series in Applied Mathematics. *SIAM, Philadelphia* **12**, (1976).

[4] Thieme, H.R. Persistence under relaxed point-dissipativity with an application to en epidemic model. *SIAM J. Math. Anal.* **24**, 407-435 (1993).

[5] Li, M.Y., Graef, J.R., Wang, L. Karsai, J. Global dynamics of a SEIR model with varying total population size, *Math. Biosci.* **160**, 191-213 (1999).

[6] Zhao, X.Q. Uniform persistence and periodic coexistence states in infinite-dimensional periodic semi flows with applications. *Can. Appl. Math. Quart.* **3**, 473-495 (1995) .

[7] Castillo-Chavez, C. & Song B. Dynamical models of tuberculosis and their applications, *Math Biosc Eng.* **1**(2), 361–404 (2004).
